# The effects of intravitreal injections on intraocular pressure and retinal nerve fiber layer: a systematic review and meta-analysis

**DOI:** 10.1038/s41598-020-70269-7

**Published:** 2020-08-06

**Authors:** Victor. A. de Vries, Fabiana L. Bassil, Wishal. D. Ramdas

**Affiliations:** Department of Ophthalmology, Erasmus Medical Center, PO Box 2040, 3000 CA Rotterdam, The Netherlands

**Keywords:** Ocular hypertension, Glaucoma, Retinal diseases

## Abstract

The number of eye diseases treated with intravitreal injections is increasing. Obviously, an injection of fluid into the eye results in an increase of intraocular pressure (IOP), the main risk factor for glaucoma. However, the effect of these repeated IOP increases on the eye is unclear. Therefore, we performed a systematic review with meta-analyses. PubMed, Embase and Clinical Trials Registries were searched for articles investigating the relationship between intravitreal injections (anti-vascular endothelial growth factor [anti-VEGF] or steroids) and either IOP, retinal nerve fiber layer (RNFL)-thickness and glaucoma. Multiple meta-analyses were performed, combining data on intravitreal injection of anti-VEGF medication and dexamethasone implants. A total of 74 articles were eligible for meta-analyses. The short-term effect of an intravitreal injection of anti-VEGF showed a statistically significant increase in IOP. One day after injection of anti-VEGF, however, IOP was significantly lower than baseline. The long-term time-intervals showed no significant difference in IOP. After intravitreal injection of a dexamethasone implant, IOP was significantly higher than baseline 1 month post-injection. RNFL-thickness was significantly reduced 6 and 12 months post-injection of anti-VEGF, as well as at end of follow up. Caution is advised when using intravitreal medication, especially when treating patients with advanced glaucoma; in these cases, prophylactic IOP-lowering medication may be considered.

## Introduction

The intravitreal administration of medication through injections has revolutionized the treatment for several retinal disorders. One of the first descriptions of an intravitreal injection was in 1911 when Ohm reported a method to inject air into the eye for retinal tamponade^1,2^. Almost half a century later, Sorsby and Ungar treated an endophthalmitis case with intravitreal administration of Penicillin^3^. Nowadays, besides antibiotics several types of intravitreal medications are available such as anti-vascular endothelial growth factor (anti-VEGF; e.g.bevacizumab, ranibizumab, and aflibercept). Since the introduction of anti-VEGF in 2004, the indications of anti-VEGF for retinal disorders have been increasing continuously^4–6^. Choroidal neovascularization is a leading cause of vision loss and can occur in a variety of retinopathies [e.g. diabetic retinopathy, age-related macular degeneration (AMD), and retinal vein occlusion]^7,8^. Currently approximately 196 million people suffer worldwide from AMD, an estimated 16.4 million suffer from some form of retinal vein occlusion, and 93 million people from diabetic retinopathy^9–11^. As a result of both population growth and increased lifespan globally, these retinopathies are only expected to become even more prevalent. For example, it is estimated that the number of people suffering from AMD will have risen to 288 million by 2040^10^. The process of angiogenesis associated with these conditions is heavily mediated by VEGF^12^. Blocking the actions of VEGF has shown to be an effective way of blocking neovascularization, substantially limiting the vision loss associated with these retinopathies^13^. As a result routine injection of anti-VEGF has been implemented on an immense scale. More recently intravitreal steroids (e.g. dexamethasone implants) have also been introduced.

In general, these injections are considered relatively safe, nevertheless, long-term use of the injections can increase the chance of ocular complications^14,15^. Obviously, if a fluid is injected into the eye the intraocular pressure (IOP) should increase. Therefore immediately after the administration of an intravitreal injection, acute fluctuations of the IOP will be present; which are related to the procedure and usually last a maximum of a few hours^14,15^. Recently however, several studies have reported that the administration of multiple intravitreal injections with anti-VEGF is associated with an increased risk for sustained IOP elevation, especially in patients with pre-existing glaucoma^16^. An increase in IOP is a major risk factor for thinning of the retinal nerve fiber layer (RNFL)^17^. Chronic suppression of VEGF may cause harmful downstream effects on the RNFL. Furthermore, research has shown that the post-injection IOP fluctuations can also be damaging to the RNFL^18^. Steroids are also notorious for their steroid-induced increase in IOP^19^.

Although a lot of research has been done on this topic, the results are not consistent. It is clearly stated in multiple research what the short-term effects are; yet the long-term results have not been definitive ^20^. A recently published report on the effect of anti-VEGF on IOP and glaucoma has shown that already much research has been done on this topic, nonetheless the results have not been concisely quantified in a meta-analysis^20^.

Therefore, we performed a systematic review and meta-analyses to address the conflicting results regarding the short- and long-term effect of intravitreal injections on IOP, RNFL-thickness, and glaucoma. Using data from both observational and interventional studies, we calculated the pooled effect size for anti-VEGF and dexamethasone both for short- and long-term outcomes. Finally, we reported the current level of evidence for a potentially negative effect of intravitreal injections on IOP, RNFL-thickness, and glaucoma.

## Methods

This systematic review was performed according to the Preferred Reporting Items for Systematic review and Meta-Analyses (PRISMA) and the Meta-analysis Of Observational Studies in Epidemiology (MOOSE) guidelines^21,22^.

### Eligibility criteria for considering studies for this review

We only included observational or interventional quantitative studies, which reported on the relationship between intravitreal injection of anti-VEGF (bevacizumab, ranibizumab, and aflibercept) or steroids (dexamethasone implant) vs. either IOP, RNFL-thickness, or glaucoma. With the goal of maximizing generalizability we did not exclude studies based on the indication(s) for treatment. In contrast to the eligibility criteria for IOP, the studies which reported mean RNFL-thickness for a patient population which received multiple types of injections, were also included. Unfortunately, our literature search yielded no studies in which glaucoma served as the outcome.

### Search methods for identifying studies

Two researchers (VAdeV and FLB) independently conducted literature searches in the PubMed, and Embase databases and Clinical Trial Registries (clinicaltrialsregister.eu; clinicaltrials.gov) on August 7th^23,24^, 2019. The following search strategy was performed to search for articles investigating the relationship between intravitreal injections and IOP: “(intravitreal bevacizumab AND intraocular pressure) OR (intravitreal ranibizumab AND intraocular pressure) OR (intravitreal aflibercept AND intraocular pressure) OR (intravitreal dexamethasone AND intraocular pressure)”. Similarly, a search was performed for studies investigating intravitreal injection and RNFL-thickness: “(intravitreal bevacizumab AND retinal nerve fiber layer) OR (intravitreal ranibizumab AND retinal nerve fiber layer) OR (intravitreal aflibercept AND retinal nerve fiber layer) OR (intravitreal dexamethasone AND retinal nerve fiber layer) OR (intravitreal AND retinal fiber layer)”. Finally, we performed a search for studies on the relationship between intravitreal injections and glaucoma: “(intravitreal injections) AND (glaucoma) NOT (neovascular glaucoma) NOT (surgery)”. We only included studies with an available abstract, with human participants, and studies of which a full text was available in either English or German.

### Study selection

From the initial search, titles and abstracts were scanned independently by two authors for eligible articles, in which the relationship between intravitreal injection and IOP or RNFL-thickness, respectively, was investigated, without any other restricting selection criteria. Next, the full text of the eligible studies was used to select additional studies from the reference lists in a similar fashion. Finally, the results were compared, and discrepancies were discussed. If the two researchers could not reach consensus, a third researcher was involved to clarify.

### Data collection and risk of bias assessment

For each study we extracted the name of the authors, title, year of publication, study design, sample size, method of assessing outcome(s), indication for injection, injected medication (exposure), means with corresponding standard deviations for both a post-injection and control group, and type of control (i.e., the same eye pre-injection, the contralateral eye, or healthy volunteers). To assess the methodological quality and risk of bias within individual studies the Newcastle–Ottawa Scale (NOS) for assessing the quality of comparative non-randomized studies was used. For randomized studies we used the Cochrane risk-of-bias Tool for Randomized Trials (RoB2). Two authors assessed the retrieved studies independently, after which any discrepancies were discussed. Where these two authors could not reach consensus, a third would give resolution. The certainty of evidence was determined by using the Grading of Recommendations, Assessment, Development, and Evaluation (GRADE) approach.

### Data synthesis and analysis

As the number of studies reporting an effect estimate of intravitreal injections on glaucoma was zero, a meta-analysis could not be performed. Therefore, we focused on the most important quantitative measures for treatment (IOP) and diagnosis (RNFL) of glaucoma. The primary outcome in the IOP-analyses and RNFL-thickness analyses was the difference in IOP and RNFL-thickness, respectively, between pre- (baseline) and post-injection (follow-up). For both primary outcomes we performed meta-analyses to calculate the weighted mean difference (WMD) with corresponding 95% confidence interval (CI) for each of the four intravitreal medications (e.g. bevacizumab, ranibizumab, aflibercept, or dexamethasone implant), and for all anti-VEGF medications combined, for all time intervals with at least 3 studies reporting IOP or RNFL. The specified time-intervals were: within 1 min after injection, 5 min after injection, 10 min, 15 min, 30 min, 1 h, 1 day, 1 week, 2 weeks, 1 month, 2 month, 3 months, 4 months, 6 months, and 12 months after injection. We also performed a meta-analysis for the RNFL-thickness at the end of follow-up. If a meaningful meta-analysis could not be conducted for one of the intravitreal medications, for example, in case of an insufficient number of studies, we reported the individual results of these studies. The threshold for statistical significance was identified as a P value of 0.05 or less.

Heterogeneity of the meta-analyses was measured by calculating I^2^^25^. In case of low heterogeneity (I^2^ < 50%), a fixed effects model was used in our meta-analyses. In case of high heterogeneity (I^2^ > 50%) a random effects model was used.

Statistical analyses were performed using Review Manager 5.3 for Windows (Copenhagen: The Nordic Cochrane Centre, The Cochrane Collaboration, 2014) and SPSS (IBM, Statistical Package for Social Sciences, Release 24.0.0.1).

## Results

Across all performed searches a total of 4,024 articles were found, of which 77 were included in our review and 74 were included in our meta-analyses.

**Figure 1:**
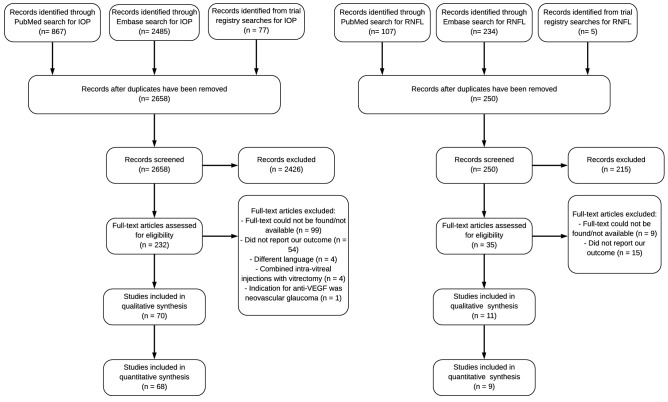
Flow diagram (according to PRISMA) showing the selection process for inclusion of studies from our searches on the relationship between intravitreal injection and IOP or RNFL-thickness. *IOP* intraocular pressure, *RNFL* retinal nerve fiber layer, *PRISMA* Preferred Reporting Items for Systematic Reviews and Meta-Analyses.

The literature search for studies investigating IOP yielded a total of 70 eligible articles^26–95^, of which 68 were eligible for meta-analyses (Fig. 1; Supplementary Table S1). The literature search for studies investigating RNFL-thickness yielded a total of 11 eligible articles^58,86,96–103^, of which 9 were eligible for meta-analyses (Fig. 1; Supplementary Table S1). Overall, the methodological quality within individual studies was good (Supplementary Tables S2, S3).

The literature search for studies investigating the relationship between intravitreal injections and glaucoma yielded 260 articles from Pubmed and 448 articles from Embase. After scanning the articles and reading the full texts of potentially suitable articles, no studies were deemed eligible for inclusion.

### Intravitreal injection of anti-VEGF and IOP

For the meta-analysis of bevacizumab, a total of 27 studies (1,488 eyes), investigating IOP after intravitreal injection were identified for meta-analysis (Supplementary Table S1; Supplementary Fig. S1). The WMD [95% CI] for IOP (in mmHg) was 23.88 [22.41, 25.34] immediately after injection and declined to 2.48 [1.24, 3.72] 30 min post-injection (Supplementary Fig. S2). On the day after injection the WMD of IOP (in mmHg) was significantly lower (− 1.19 [− 2.00, − 0.37]) compared to baseline, and no significant difference in IOP was found for any later time-interval. For ranibizumab, 14 studies (1,057 eyes) were included for the meta-analysis, which reported on IOP after intravitreal injection (Supplementary Table S1, Supplementary Fig. S3). The WMD [95% CI] in IOP (in mmHg) was 25.95 [21.46, 30.45] immediately after injection and declined to 2.61 [1.76, 3.46] 30 min post-injection (Supplementary Fig. S4). Although the IOP showed a downwards trend on the day after injection with an WMD of − 0.17 [− 0.59, 0.26], this difference was not statistically significant (*P* = 0.44). The WMD of IOP was also not significantly different than baseline after the later time-intervals. For aflibercept, 4 studies (133 eyes) were found (Supplementary Table S1). The minimum threshold of 3 studies or more was not reached for a single time-interval. Therefore, a meaningful meta-analysis could not be performed on the relationship between aflibercept intravitreal injections and post-injection IOP. Studies reporting on aflibercept were, however, included in our meta-analysis on IOP after intravitreal injection of any anti-VEGF (i.e., all anti-VEGF medications combined).

**Figure 2:**
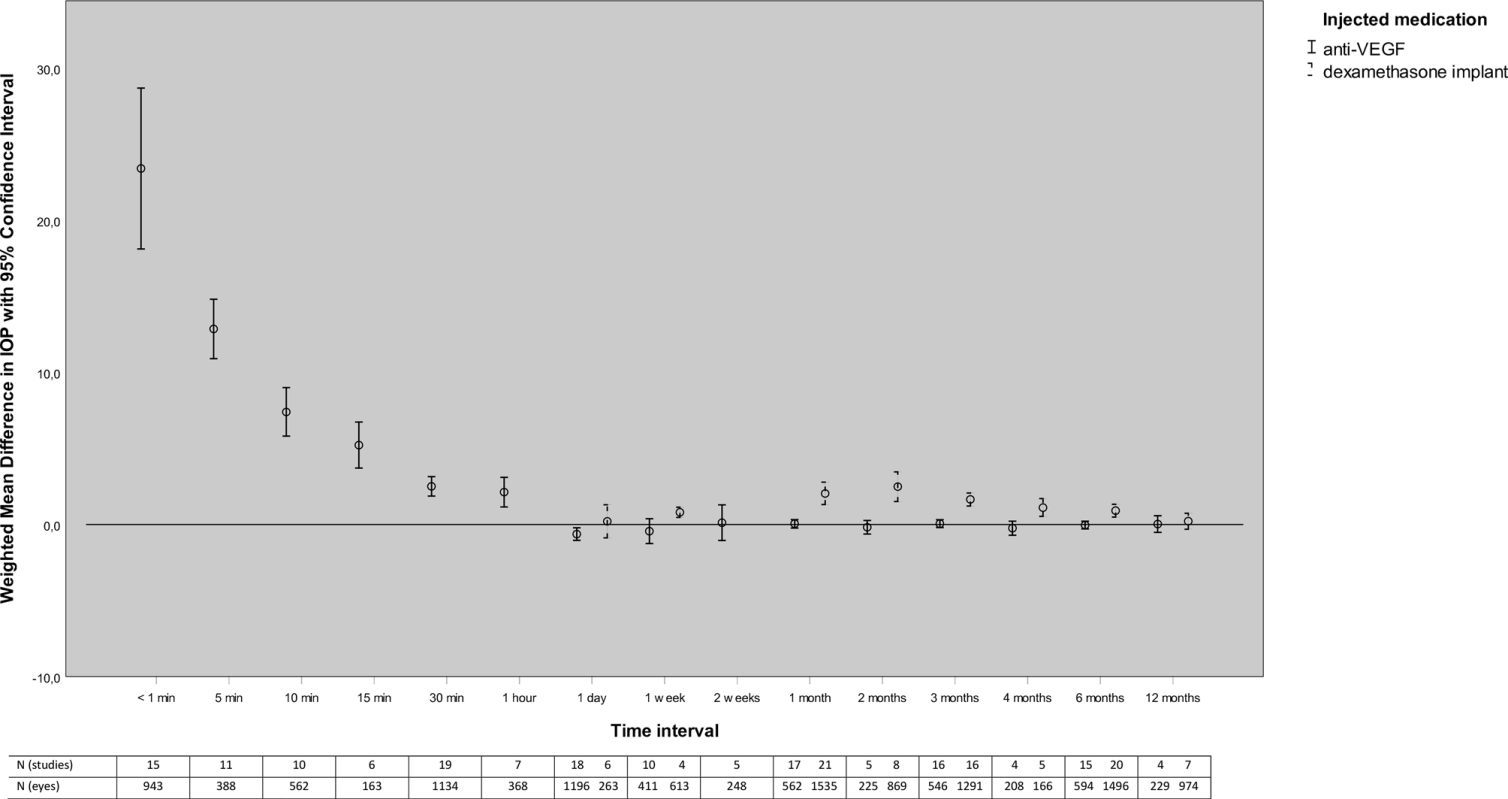
Weighted mean difference in IOP during follow-up after intravitreal injection of anti-VEGF and Dexamethasone implant. *IOP* intraocular pressure, *VEGF* vascular endothelial growth factor.

A total of 46 articles (2,872 eyes) were included for the meta-analyses on the relationship between the intravitreal injection of any anti-VEGF and post-injection IOP. The meta-analyses were performed for 15 different time intervals, ranging from the IOP immediately after injection (< 1 min) to the IOP after 12 months (Fig. 2; Supplementary Fig. S5). On the day of injection, we found for all included time-intervals a statistically significant increase in IOP. The WMD [95% CI] for IOP (in mmHg) was 23.41 [18.12, 28.70] immediately after injection and declined to 2.51 [1.87, 3.15] after 30 min. On the day after injection of anti-VEGF, however, the WMD [95% CI] for IOP (in mmHg) was significantly lower at − 0.63 [− 1.04, − 0.22]. IOP normalized after one week to 0.12 [− 1.05, 1.29] and no significant difference in IOP compared to baseline was found for all subsequent intervals. Heterogeneity (I^2^) was higher than 50% for all our short-term time-intervals up to and including IOP at 2 weeks post-injection. For IOP at 1-month post-injection and all later time-intervals (2, 3, 4, 6, and 12 months post-injection) heterogeneity was below 50%.

### Intravitreal injection of a dexamethasone implant and IOP

A total of 26 articles (1,744 eyes) on intravitreal injection of a dexamethasone implant and IOP were identified for meta-analysis (Fig. 2; Supplementary Fig. S6). None of the identified studies reported on the change in IOP within 1 day after injection. No statistically significant difference was found between the IOP 1 day after injection and IOP at baseline (*P* = 0.70). IOP was significantly increased at all later time-intervals, except of at end of follow-up (12 months: *P* = 0.11). The WMD [95% CI] for IOP (in mmHg) was 0.80 [0.47, 1.14] at 1 week, 2.04 [1.31, 2.78] at 1 month, 2.49 [1.51, 3.46] at 2 months, 1.65 [1.22, 2.07] at 3 months, 1.11 [0.53, 1.70] at 4 months, and 0.91 [0.49, 1.33] at 6 months after intravitreal injection. Heterogeneity (I^2^) was higher than 50% for all specified time-intervals, except for 1 week (I^2^ = 0%) and 12 months post-injection (I^2^ = 38%).

### Intravitreal injection of anti-VEGF or dexamethasone implant and RNFL-thickness

Our literature search yielded 11 articles reporting on the relationship between anti-VEGF or dexamethasone implants and the average RNFL-thickness.

**Figure 3:**
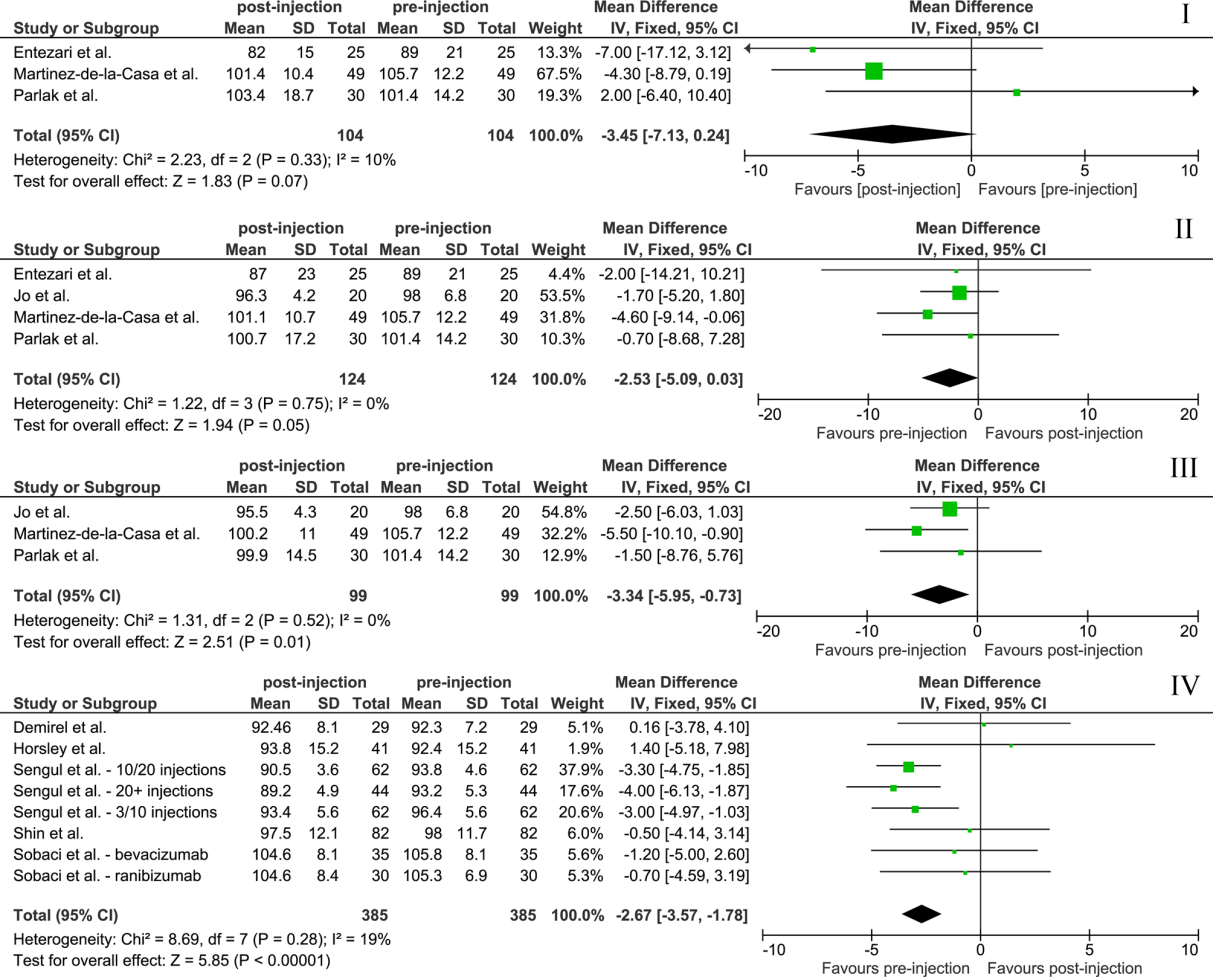
Meta‐analyses for the association of anti-VEGF and RNFL-thickness. Black diamonds indicate the overall WMD. The size of the gray box is inversely proportional to the variance. Horizontal lines indicate 95% CI. The vertical line in each panel shows the value for no effect (WMD = 0). *RNFL* retinal nerve fiber layer, *WMD* weighted mean difference, *I* 3 months post-injection, *II* 6 months, *III* 12 months, *IV* at end of follow-up.

Five studies reported on RNFL-thickness at end of follow-up and were eligible for meta-analyses (Supplementary Table S1). Mean ± standard deviation follow-up for studies included in the meta-analysis was 23.40 ± 9.08 months. A total of 385 eyes were included in this meta-analysis. The WMD [95% CI] was − 2.67 [− 3.57, − 1.78] µm (Fig. 3).

Six studies investigated RNFL-thickness at a specific point in time during follow-up. Bulut et al. found that 6 months after dexamethasone implant the average RNFL-thickness had been reduced from 104.1 ± 11.1 µm to 101.1 ± 10.4 µm (*P* < 0.001)^37^. Four of the studies (124 eyes) were included in our meta-analysis for anti-VEGF. The WMD [95% CI] for RNFL-thickness (in µm) at 3, 6, and 12 months was − 3.45 [− 7.13, 0.24], − 2.53 [− 5.09, 0.03], and − 3.34 [− 5.95, − 0.73], respectively. RNFL-thickness was only significantly reduced at 12 months (*P* = 0.01; Fig. 3). Heterogeneity (I^2^) was low between 0 and 19% for the specified time-intervals.

### Risk of bias and overall certainty of evidence

The mean ± standard deviation Newcastle–Ottawa sum score for all observational studies included in this review was 8.65 ± 0.66 (Supplementary Table S2). For observational studies investigating IOP and RNFL-thickness the score was 8.64 ± 0.69 and 8.77 ± 0.43, respectively. The Cochrane risk-of-bias for randomized studies tool showed a high risk of bias for all interventional studies (Supplementary Table S3). As per the GRADE approach, the evidence of this meta-analysis warrants a low level of certainty (Supplementary Table S4).

## Discussion

### Summary of findings

In this study we summarized the available scientific literature on the effect of anti-VEGF intravitreal injections and dexamethasone implant injections on the most important quantifiable measures for glaucoma (IOP and RNFL-thickness). After intravitreal injection of anti-VEGF, the IOP was significantly increased for all measured time-intervals on the day of injection. IOP was slightly decreased on the day after injection. IOP did not differ significantly from baseline during any later follow-up measurements. After the injection of a dexamethasone implant, IOP was increased for all long-term time-intervals until the 12-month endpoint.

Substantially less evidence was available on the relationship between intravitreal injections and RNFL-thickness. For our meta-analysis pooling all anti-VEGF results at end of follow-up we found a significant reduction in RNFL-thickness at 12 months and at end of follow-up. However, with a RNFL-thickness reduction of only 2 to 3 µm after an average follow-up of 23 months, the clinical relevance of these findings is unclear.

### Relationship with literature

As mentioned before, the IOP showed a slight decrease the day immediately after injection, according to research this is most likely due to ciliary body inflammation as a result of the sharp IOP rise after the intravitreal injection^76^.

Although the literature on alternative corticosteroids is still sparse, some studies have shown that triamcinolone acetonide intravitreal injections result in similar rates of ocular hypertension compared to dexamethasone implant^104,105^. Similarly Vaz-Pereira et al. could not find a significant difference in IOP after switching from dexamethasone to fluocinolone acetonide^106^. More research will have to be done in order to be able to more clearly distinguish the pros and cons of the different corticosteroid injections available.

There are several known risk factors for glaucoma such as older age, a family history of glaucoma, and myopia^107–109^. It has been suggested that the recurrent transient rising IOP after intravitreal injection could also be correlated with an increased incidence of glaucoma. A recent report by the American Academy of Ophthalmology reviews this effect and found mixed results, with 6 studies reporting an incidence between 4 and 15% of sustained IOP elevation and 7 studies reporting no long-term change in IOP^20^. By performing meta-analyses, we have come to a more definitive answer: IOP normalizes within days after intravitreal injection of anti-VEGF, but after injection of a dexamethasone implant IOP remains elevated. Recurrent injections of anti-VEGF reduce RNFL-thickness; however, this reduction does not appear to be clinically relevant. Moreover, the studies used different optical coherence tomography (OCT)-devices of which the results may not be interchangeable^110^. However, within the studies baseline and follow-up RNFL were measured with the same OCT-device.

Several studies have shown that the post-intravitreal injection rise in IOP can be prevented by using glaucoma medication such as brimonidine/timolol and acetazolamide. For example, administering patients 500 mg oral acetazolamide 60–90 min before injection resulted in a significant reduction in IOP at 30 min post-injection of 5.5 mmHg^74^. Another study evaluated the effects of oral acetazolamide and topical brimonidine tartrate. In this study the IOP of pretreated eyes returned to baseline within 10 min, whereas eyes that were not pretreated remained elevated at 30 min post-injection^111^. A fixed combination of brimonidine/timolol twice a day on the day before and the day of the intravitreal injection was also an effective prophylaxis to reduce the acute IOP-spikes of the post-injection period^112^. At 5 min post-injection the mean IOP was significantly higher in the control group when compared with the pretreatment group. The mean IOP normalized at 15 min for the pretreatment group, whereas this was only the case after 30 min for the control group^112^. Additionally, several studies have shown that prophylactic anterior chamber paracentesis is a safe and effective option for preventing IOP spikes after an intravitreal injection^87,113,114^. Most notably, Enders et al. found a significant RNFL-loss in patients who had not undergone anterior chamber paracentesis prior to their intravitreal injections, but could not find a significant reduction in RNFL-thickness in those who did^115^. We can therefore conclude that there are several effective options for IOP prophylaxis. It has been suggested that the acute IOP changing after intravitreal injections could be causing perfusion-related injury to ocular structures, potentially resulting in glaucomatous damage to the macula and optic nerve^116^. This mechanism may be similar to the IOP-increase seen during intraocular surgery, which may result in a sudden loss of vision, also known as “wipe-out”^117,118^. During cataract surgery, which takes around 7 min, IOP increases up to 30–40 mmHg several times. This is one of the main reasons that ophthalmologists are very reluctant to operate on eyes with advanced glaucoma (visual field < 10°). A similar rise in IOP is seen after intravitreal injections, which therefore might have a similar effect of visual field loss. Scientific evidence to support this hypothesis is nonetheless lacking^119^.

IOP monitoring and prophylaxis should therefore be considered before giving intravitreal injections, especially in patients with advanced glaucoma or patients at risk for glaucoma who will receive a dexamethasone implant.

### Strengths and weakness

The strengths of this study include a high quality and number of reviewed studies. By comparing the post-injection IOP or RNFL-thickness with the pre-injection IOP or RNFL-thickness of the same eye, the chance of confounding factors influencing the outcome was minimalized. Furthermore, we performed a large amount of meta-analyses to combine all available evidence in a concise and clear manner. By looking at both IOP and RNFL-thickness, we attempted to establish both the development of IOP over time after an intravitreal injection, and the long-term effect of these IOP elevations. Finally, by including studies regardless of the indication for an intravitreal injection, our results are generalizable, and its implications can be applied to a large number of patients. For example, most patients treated with anti-VEGF are treated with different types of anti-VEGF during their life depending on the effect of the anti-VEGF on the indicated disease.

Nevertheless, some limitations have to be considered. Most notably, the heterogeneity was high for some of our IOP related meta-analyses and especially for our short-term time-intervals. This is possibly due to the volatile and unpredictable nature of the immediate IOP rise after injection. Alternatively, the variability in IOP-measuring methods, frequency of injection, and the usage of IOP-lowering agents could have an effect on the heterogeneity of this review. During a previous study a greater IOP elevation post-injection was seen, if the interval between injections was less than 8 weeks and the frequency of injections in eyes with a sustained IOP elevation was significantly higher than in eyes without sustained IOP elevation^120^. Another factor that could contribute to the heterogeneity is the type of anti-VEGF medication used. We included studies evaluating the effect of either bevacizumab, ranibizumab or aflibercept. According to previous research, a significant IOP increase can be found when using bevacizumab and ranibizumab and not with aflibercept^121–124^. We did not exclude studies based on the use of anesthetic method for the intravitreal injection. One study has shown that decompressing the eye with lidocaine soaked cotton swabs applied with moderate pressure, compared to lidocaine gel applied without pressure, reduces the post-injection IOP spike^52^. A last factor that could explain the heterogeneity is that some studies did not exclude patients with a history of glaucoma. Several studies have shown a relationship between a history of glaucoma and higher IOP elevations after injection^122,125,126^.

Secondly, this systematic review is based primarily on observational studies, with only a view RCT’s included. Some unknown confounding or predisposing factors may have been missed; a threat inherent to the observational nature of these studies.

Thirdly, although the injection of a dexamethasone implant did seem to result in a sustained rise in IOP, we were not able to evaluate the long-term effects of this elevation on RNFL-thickness, due to a lack of available literature on this topic.

### Conclusion and recommendation

IOP rises sharply immediately after intravitreal injection of anti-VEGF. This appears to be a transient rise, with the IOP normalizing within 1–7 days. However, after the injection of a dexamethasone implant, IOP will remain elevated for several months, before finally normalizing 1-year post-injection. Caution is therefore advised with the use of this form of intravitreal medication. Very little evidence is available on the long-term effects on RNFL-thickness. Some diminishment in RNFL-thickness seems to occur after intravitreal injection of anti-VEGF, but the clinical relevance of this finding remains unclear. In particular, the relationship between the intravitreal injection of a dexamethasone implant and RNFL-thickness requires further research. The current results suggest that the effect of intravitreal injection on glaucoma is not of clinical significance, however, caution should be taken when treating patients with advanced glaucoma. In these cases, prophylactic IOP-lowering medication or anterior chamber paracentesis should be considered. Future studies with visual field progression or glaucoma as an outcome itself are warranted.

## Supplementary information

Supplementary file1
