# Supplementary material for: The effects of intravitreal injections on intraocular pressure and retinal nerve fiber layer: a systematic review and meta-analysis
Source: Sci Rep. 2020 Aug 6;10:13248. doi: 10.1038/s41598-020-70269-7 (PMC7411061; doi:10.1038/s41598-020-70269-7)
Supplement: Supplementary file 1 — Supplementary file1 [file 41598_2020_70269_MOESM1_ESM.pdf]

Supplementary appendix

The effects of intravitreal injections on intraocular pressure and retinal nerve fiber layer: a systematic review and meta-analysis

Contents:

TABLE S1: Summary of the retrieved studies from the systematic review

TABLE S2: Quality assessment of the included non-randomized studies according to the Newcastle-Ottawa Scale

TABLE S3: Quality assessment of the included randomized studies according to the Cochrane Risk of Bias Tool for Randomized Controlled Trials

TABLE S4: Assessment of Certainty of Evidence using Grading of Recommendations, Assessment, Development, and Evaluation

FIGURE S1: Weighted mean difference in intraocular pressure during follow-up after bevacizumab intravitreal injection.

FIGURE S2: Meta-analyses for the association of bevacizumab with IOP.

FIGURE S3: Weighted mean difference in intraocular pressure during follow-up after ranibizumab intravitreal injection.

FIGURE S4: Meta-analyses for the association of ranibizumab with IOP.

FIGURE S5: Meta-analyses for the association of anti-VEGF with IOP.

FIGURE S6: Meta-analyses for the association of dexamethasone implant with IOP.

TABLE S1. Summary of the retrieved studies from the systematic review

STUDY	STUDY DESIGN	INDICATION(S)	METHOD USED FOR OUTCOME MEASUREMENT	N (eyes)	INTRAVITREAL INJECTION TYPE(S)	EYES RECEIVING IOP-LOWERING TREATMENT (%)
**IOP**						
Alagöz et al.	prospective cohort	ME secondary to DRP, RVO	Tono-Pen applanation	34	dexamethasone implant	0%
Alba-Linero et al.	prospective cohort	uveitis	N/A	79	dexamethasone implant	31.5%
Ali et al.	RCT	DME	Goldmann applanation	30	dexamethasone implant	0%
Arikan et al.	prospective cohort	AMD, DRP, RVO, cystoid ME, other (unspecified)	Perkins hand-held tonometer	106	ranibizumab	N/A
Arikan Yogun et al.	retrospective cohort	DME	N/A	41	dexamethasone implant	33%
Arrascue et al.	retrospective cohort	ME	N/A	37	dexamethasone implant	56.7%
Ayar et al.	retrospective cohort	BRVO	N/A	26	dexamethasone implant	38%
Al-Khersan et al	retrospective cohort	DME	Goldmann applanation	17	dexamethasone implant	82.4%
Bansal et al.	retrospective cohort	recalcitrant DME	Goldmann applanation	67	dexamethasone implant	12%
Bellocq et al. (2015)	retrospective case series	ME	applanation (unspecified)	50	dexamethasone implant	20%
Bellocq et al. (2017)	retrospective case series	ME	applanation (unspecified)	100	dexamethasone implant	21%
Bulut et al.	prospective cohort	ME secondary to BRVO	appalanation (unspecified)	33	dexamethasone implant	N/A
Cacciamani et al.	prospective cohort	nAMD	Tono-Pen applanation	25	bevacizumab	4%
Choi et al.	retrospective cohort	ME	NCT or Goldmann applanation	540	dexamethasone implant	60%
Coskun et al.	retrospective cohort	active Behçet posterior uveitis	Goldmann applanation	17	dexamethasone implant	17%
Cunha et al.	clinical trial	BRVO, CRVO	Goldmann applanation	10	dexamethasone implant	30%
Demirel et al.	retrospective cohort	RVO, DME, AMD	NCT	202	ranibizumab	0.5%
Ding et al.	RCT	ME	Goldmann applanation	16	bevacizumab	0%
el-Chehab et al.	prospective cohort	wet AMD	Perkins tonometer	30	aflibercept	N/A
Faghihi et al.	prospective case series	bilateral naive DME	N/A	64	bevacizumab	N/A

Falkenstein et al.	RCT	nAMD	Goldmann applanation / TonoPen	70	bevacizumab	N/A
Frère et al.	retrospective cohort	active noninfectious uveitis	applanation (unspecified)	20	dexamethasone implant	49.6%
Fuest et al.	prospective cohort	ME due to either wet AMD, DM or RVO	rebound tonometry (i-care)	29	ranibizumab	N/A
Gado et al.	RCT	CRVO with ME	Goldmann applanation	30	bevacizumab, dexamethasone implant	20%
Gismondi et al.	prospective cohort	nAMD	Tono-Pen applanation	54	ranibizumab	0%
Goktas et al.	prospective cohort	wet-type subfoveal/juxtafoveal choroidal neovascular membrane	NCT	31	ranibizumab	N/A
Gregori et al.	RCT	various indications (unspecified)	Tono-Pen applanation	48	ranibizumab	N/A
Guler et al.	clinical trial	AMD, CRVO, BRVO, DME, diabetic vitreous hemorrhage, retinal artery macroaneurysm, CSCR	Goldmann applanation	43	bevacizumab	N/A
Higashiyama et al.	RCT	ME secondary to BRVO	N/A	22	bevacizumab	0%
Hohn et al.	prospective cohort	AMD	Schiotz tonometry	31	ranibizumab	N/A
Hollands et al.	prospective cohort	nAMD, glaucoma, DME, other (unspecified)	Goldmann applanation	104	bevacizumab	1%
Jaffar et al.	prospective cohort	DME	Goldmann applanation	100	bevacizumab	N/A
Jo et al.	prospective cohort	unilateral exudative AMD	Goldmann applanation	20	ranibizumab	N/A
Kaldirim et al.	retrospective cohort	ME secondary to BRVO	Goldmann applanation	22	ranibizumab, aflibercept, dexamethasone implant	25%
Karakurt et al.	retrospective cohort	AMD, CRVO, BRVO or DME	Icare rebound tonometry	81	ranibizumab, aflibercept, dexamethasone implant	N/A
Kernt et al.	case series	unspecified	Goldmann applanation	45	bevacizumab	0%
Kim, D et al.	retrospective cohort	AMD	NCT	83	bevacizumab	3.6%
Kim, GN et al.	clinical trial	AMD CNV, idiopathic CNV, RVO, DRP, myopic CNV	pneumatomometer	10	bevacizumab, ranibizumab	0%
Kim, JY et al.	retrospective cohort	ME from BRVO	Goldmann applanation	22	bevacizumab	N/A
Lee, K et al.	clinical trial	CNV, RVO, other (unspecified)	Goldmann applanation	61	bevacizumab	N/A
Lee, JW et al.	prospective cohort	DME, PDR, AMD, RVO related ME, CSC, ICNV	Icare rebound tonometry	65	anti-VEGF	N/A
Lemos et al.	prospective cohort	ME or active choroidal neovascularization	Goldmann applanation	106	bevacizumab	20.8%
Lemos-Reis et al.	prospective cohort	AMD, myopia, DME, BRVO, pseudoxanthoma	Icare rebound tonometry	291	bevacizumab	N/A

		elasticum, uveitis, idiopathic				
Lim et al.	prospective cohort	AMD, RVO, DRP, CSCR	Tono-Pen applanation	25	bevacizumab	N/A
Marey et al.	prospective cohort	DRP	N/A	30	bevacizumab	0%
Mazzarella et al.	retrospective cohort	ME	Goldmann applanation	65	dexamethasone implant	12.3%
Mazzulla et al.	retrospective cohort	unspecified	Goldmann applanation	29	bevacizumab	0%
Mishra et al.	RCT	ME secondary to CRVO	Goldmann applanation	20	dexamethasone implant	25%
Murray et al.	RCT	nAMD	Tono-Pen applanation	12	ranibizumab	N/A
Nalcaci et al.	retrospective cohort	DME	N/A	20	dexamethasone implant	25%
Omay et al.	prospective cohort	exudative AMD, ME secondary to RVO/DM/CSCR	NCT	97	bevacizumab, ranibizumab	N/A
Özata et al.	retrospective cohort	persistent DME	Goldmann applanation	50	dexamethasone implant	10%
Ozkan et al.	retrospective cohort	ME	air tonometry	21	bevacizumab	N/A
Özkaya et al.	RCT	AMD	Goldmann applanation	35	ranibizumab	N/A
Park et al.	prospective cohort	BRVO, AMD, DRP, CRVO, DME	NCT (rebound tonometer where NCT not possible)	42	bevacizumab	N/A
Pohlmann et al.	prospective cohort	noninfectious uveitis	N/A	109	dexamethasone implant	4.5%
Ramezani et al.	RCT	CRVO	Goldmann applanation	43	bevacizumab	0%
Rebolleda et al.	prospective cohort	nAMD	Perkins tonometer	30	aflibercept	N/A
Rensch et al.	clinical trial	non-ischaemic CRVO	Goldmann applanation	25	bevacizumab	N/A
Ryder et al.	retrospective cohort	noninfectious posterior uveitis, ME secondary to RVO	N/A	22	dexamethasone implant	18.2%
Sengul et al.	clinical trial	nAMD	Tono-Pen applanation	62	ranibizumab	N/A
Soheilian et al.	RCT	NPDR with ME, wet-type AMD	Tono-Pen applanation	44	bevacizumab	N/A
Song JH et al.	retrospective cohort	DME	Tono-Pen applanation	38	bevacizumab	0%
Song, S et al.	prospective case control	exudative AMD, DME, RVO, pathologic myopia, idiopathic CNV, cystoid ME	NCT	203	ranibizumab	N/A
Tas et al.	retrospective cohort	diffuse or cystoid DME	Goldmann applanation	40	bevacizumab	0%
Totan et al.	prospective cohort	DME	Tono-Pen applanation	30	bevacizumab	13.3%
Tsai et al.	retrospective cohort	ME secondary to BRVO/CRVO	NCT	23	bevacizumab	N/A
Unsal et al.	retrospective cohort	DME	Goldmann applanation	46	dexamethasone implant	32%

Yilmaz et al.	prospective cohort	DME, ME secondary to RVO	Pascal tonometry	34	dexamethasone implant	N/A
Zarranz-Ventura et al.	retrospective cohort	noninfectious uveitis with vitritis or CME or both	N/A	146	dexamethasone implant	42.6%
**RNFL**						
Bulut et al.	prospective cohort	ME secondary to BRVO	peripapillary scan, using and undefined OCT device	33	dexamethasone implant	
Demirel et al.	retrospective cohort	AMD	papillary blockscan + peripapillary nasal sector scan, using Cirrus OCT	29	ranibizumab	
Enders et al.	retrospective cohort	CNV secondary to AMD	peripapillary scan, using Spectralis OCT	32	anti-VEGF (unspecified)	
Entezari et al.	prospective cohort	neovasculair AMD	peripapillary scan, using Spectral OCT	25	bevacizumab	
Horsley et al.	retrospective cohort	AMD	peripapillary scan using an undefined OCT device	41	anti-VEGF (unspecified)	
Hwang et al.	retrospective case-control	DRP (with and without DME)	peripapillary scan + papillary blockscan + macular blockscan, using Spectralis OCT	42	bevacizumab	
Jo et al.	prospective cohort	unilateral exudative AMD	peripapillary scan + papillary blockscan, using Cirrus OCT	20	ranibizumab	
Martinez-de-la-Casa et al.	prospective cohort	neovascular AMD	peripapillary scan + macular blockscan, using Spectralis OCT	49	ranibizumab	
Parlak et al.	prospective cohort	exudative AMD	peripapillary scan, using Spectralis OCT	30	ranibizumab	
Saatci et al	retrospective cohort	NAION	peripapillary scan, using Heidelberg Spectralis OCT	17	ranibizumab	
Sengul et al.	clinical trial	nAMD	peripapillary scan, using an Optuvec OCT device	168	ranibizumab	
Shin et al.	retrospective cohort	ME secondary to AMD, DMR or RVO	peripapillary scan + macular blockscan, using	82	ranibizumab, bevacizumab	

			Spectralis OCT			
Sobaci et al.	retrospective cohort	wet AMD	peripapillary scan, using Stratus OCT	65	bevacizumab, ranibizumab	

IOP = Intraocular pressure; ME = macular edema; DRP = diabetic retinopathy; RVO = retinal vein occlusion; DME = diabetic macular edema; AMD = age-related macular degeneration; BRVO = branch retinal vein occlusion; CRVO = central retinal vein occlusion; nAMD = neovascular age-related macular degeneration; CNV = choroidal neovascularization; CSCR = central serous chorioretinopathy; PDR = proliferative diabetic retinopathy; NPDR = non-proliferative diabetic retinopathy; ICNV = idiopathic choroidal neovascularization; CSC = central serous retinopathy; NAION = non-arteritic anterior ischemic optic neuropathy; NCT = non-contact tonometer; RNFL = retinal nerve fiber layer; OCT = optical coherence tomography; RCT = randomized controlled trial

TABLE S2. Quality assessment of the included non-randomized studies according to the Newcastle-Ottawa Scale*

Author	Design	Selection	Comparability	Outcome (cohort)/ Exposure (case-control)	Total score (max.9)
Alagöz et al.	prospective cohort	4	2	3	9
Alba-Linero et al.	prospective cohort	4	2	2	8
Arikan G et al.	prospective cohort	4	2	2	8
Arikan Yogun et al.	retrospective cohort	4	2	2	8
Arrascue et al.	retrospective cohort	4	1	3	8
Ayar et al.	retrospective cohort	4	2	2	8
Al-Khersan et al.	retrospective cohort	4	2	2	8
Bansal et al.	retrospective cohort	4	2	3	9
Bellocq et al. (2015)	retrospective case series	4	2	3	9
Bellocq et al. (2017)	retrospective case series	4	2	3	9
Bulut et al.	prospective cohort	4	2	3	9
Cacciamani et al.	prospective cohort	4	2	3	9
Choi et al.	retrospective cohort	4	2	3	9
Coskun et al.	retrospective cohort	4	2	3	9
Cunha et al.	clinical trial	4	2	3	9
Demirel et al.	retrospective cohort	4	2	3	9
el-Chehab	prospective cohort	4	2	3	9
Enders et al.	retrospective cohort	4	2	3	9
Entezari et al.	prospective cohort	4	2	3	8
Faghihi et al.	prospective case series	3	2	3	8
Frère et al.	retrospective cohort	4	2	2	8
Fuest et al.	prospective cohort	4	2	3	9
Gismondi et al.	prospective cohort	4	2	3	9
Goktas et al.	prospective cohort	4	2	3	9
Guler et al.	clinical trial	4	2	3	9
Hohn et al.	prospective cohort	4	2	3	9
Hollands et al.	prospective cohort	4	2	3	9
Horsley et al.	retrospective cohort	4	2	3	9
Hwang et al.	retrospective case-control	3	2	3	8
Jaffar et al.	prospective cohort	4	2	3	9
Jo et al.	prospective cohort	4	2	3	9
Kaldirim et al.	retrospective cohort	4	2	2	8
Karakurt et al.	retrospective cohort	4	2	3	9

Kernt et al.	case series	3	2	2	7
Kim, D et al.	retrospective cohort	4	2	3	9
Kim, GN et al.	clinical trial	3	2	3	8
Kim, JY et al.	retrospective cohort	4	2	3	9
Lee, JW et al.	prospective cohort	4	2	3	9
Lee, K et al.	clinical trial	4	2	3	9
Lemos et al.	prospective cohort	4	2	3	9
Lemos-Reis et al.	prospective cohort	4	2	3	9
Lim et al.	prospective cohort	4	2	3	9
Marey et al.	prospective cohort	4	2	3	9
Martinez-de-la-Casa et al.	prospective cohort	4	2	3	9
Mazzarella et al.	retrospective cohort	3	2	3	8
Mazzulla et al.	retrospective cohort	3	1	2	6
Nalcaci et al.	retrospective cohort	4	2	3	9
Omay et al.	prospective cohort	4	2	3	9
Özata et al.	retrospective cohort	4	2	3	9
Ozkan et al.	retrospective cohort	4	2	3	9
Park et al.	prospective cohort	4	2	3	9
Parlak et al.	prospective cohort	4	2	3	9
Pohlmann et al.	prospective cohort	4	2	3	9
Rebolleda et al.	prospective cohort	4	2	3	9
Rensch et al.	clinical trial	4	2	3	9
Ryder et al.	retrospective cohort	3	1	2	6
Saatci et al	retrospective cohort	4	2	2	8
Sengul et al.	prospective cohort	4	2	3	9
Shin et al.	retrospective cohort	4	2	3	9
Sobaci et al.	retrospective cohort	4	2	3	9
Song JH et al.	retrospective cohort	4	2	3	9
Song, S et al.	prospective case control	4	2	3	9
Tas et al.	retrospective cohort	4	2	3	9
Totan et al.	prospective cohort	4	2	3	9
Tsai et al.	retrospective cohort	4	2	3	9
Unsal et al.	retrospective cohort	4	2	3	9
Yilmaz et al.	prospective cohort	4	2	2	8
Zarranz-Ventura et al.	retrospective cohort	4	2	2	8

* For each of the sections Selection, Comparability, and Outcome/Exposure, a maximum of 4, 2, and 3 points could be given, respectively. Thus, the maximum score was 9. A higher score means higher quality.

TABLE S3. Quality assessment of the included randomized studies according to the Cochrane Risk of Bias Tool for Randomized Controlled Trials*

Author	Design	Random sequence generation	Allocation Concealment	Blinding of participants and personnel	Blinding of outcome assessment	Incomplete outcome data	Selective reporting	Other bias
Ali et al.	RCT	?	?	?	?	+	+	?
Ding et al.	RCT	?	?	?	?	+	+	+
Falkenstein et al.	RCT	-	?	?	?	+	+	?
Gado et al.	RCT	+	+	+	?	?	+	+
Gregori et al.	RCT	?	?	?	?	+	+	+
Higashiyama et al.	RCT	+	+	+	?	+	+	+
Mishra et al.	RCT	?	?	?	?	+	+	+
Murray et al.	RCT	+	+	+	?	-	+	+
Özkaya et al	RCT	-	?	?	?	+	+	+
Ramezani et al.	RCT	+	-	?	?	+	+	+
Soheilian et al.	RCT	?	?	?	?	+	+	+

*RCT = randomized controlled trial

TABLE S4. Assessment of Certainty of Evidence using Grading of Recommendations, Assessment, Development, and Evaluation*

Certainty Assessment							
**No. of Studies**	**Study Design**	**Risk of Bias**	**Inconsistency**	**Indirectness**	**Imprecision**	**Publication bias**	**Certainty**
Intravitreal injection of anti-VEGF and IOP							
39	Observational studies	Not serious	Not serious	Not serious	Not serious	Not serious	⊕⊕◯◯ low
7	Interventional studies	Serious	Not serious	Not serious	Not serious	Not serious	⊕⊕⊕◯ moderate
Intravitreal injection of a dexamethasone implant and IOP							
24	Observational studies	Not serious	Serious	Not serious	Not serious	Not serious	⊕◯◯◯ very low
3	Interventional studies	Serious	Serious	Not serious	Not serious	Not serious	⊕⊕◯◯ low
Intravitreal injection of anti-VEGF or dexamethasone implant and RNFL							
13	Observational studies	Not serious	Not serious	Not serious	Not serious	Not serious	⊕⊕◯◯ low

*GRADE Working Group grades of evidence: High quality: further research is very unlikely to change our confidence in the estimate of effect; Moderate quality: further research is likely to have an important impact on our confidence in the estimate of effect and may change the estimate; Low quality: further research is very likely to have an important impact on our confidence in the estimate of effect and is likely to change the estimate; Very low quality: we are very uncertain about the estimate

FIGURE S1. Weighted mean difference in intraocular pressure during follow-up after bevacizumab intravitreal injection

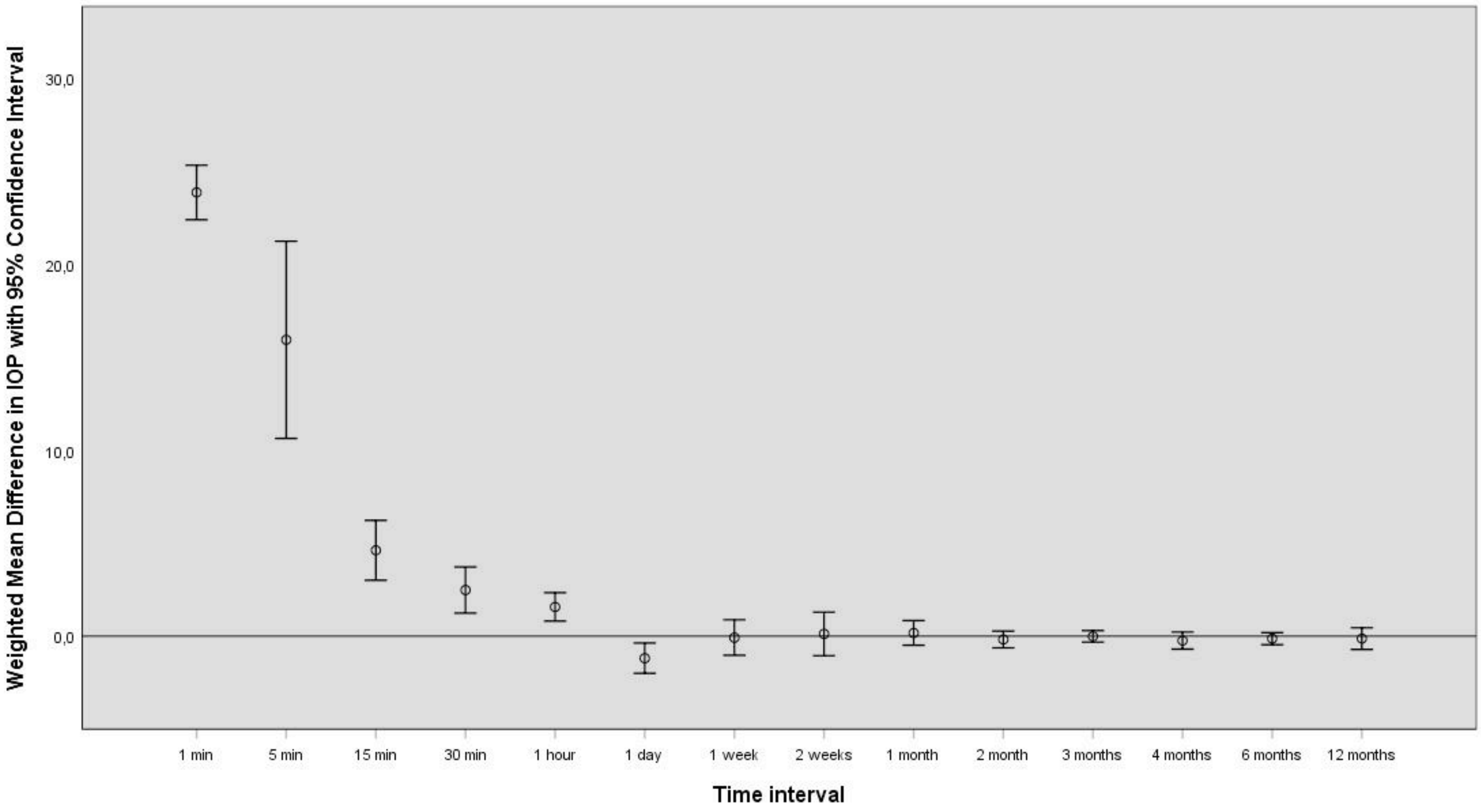

IOP = intraocular pressure

FIGURE S2. Meta-analyses for the association of bevacizumab and IOP.

I

Study or Subgroup	post-injection Mean	SD	Total	pre-injection Mean	SD	Total	Weight	Mean Difference IV, Fixed, 95% CI
Cacciamani et al.	38.44	7.92	25	16.52	3.32	25	18.9%	21.92 [18.55, 25.29]
Lemos-Reiss et al.	42.1	14.5	291	18	5.9	291	66.3%	24.10 [22.30, 25.90]
Lim et al.	43.1	9.4	25	17.7	2.5	25	14.8%	25.40 [21.59, 29.21]
**Total (95% CI)**			**341**			**341**	**100.0%**	**23.88 [22.41, 25.34]**

Heterogeneity: Chi² = 1.97, df = 2 (P = 0.37); I² = 0%
Test for overall effect: Z = 31.95 (P < 0.00001)

Mean Difference IV, Fixed, 95% CI

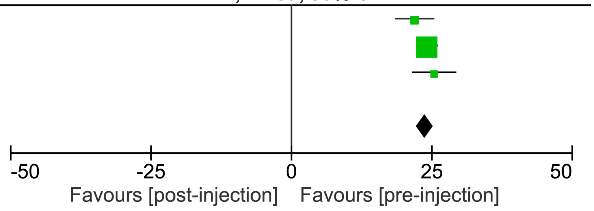

II

Study or Subgroup	post-injection Mean	SD	Total	pre-injection Mean	SD	Total	Weight	Mean Difference IV, Random, 95% CI
Kim, GN et al. - bevacizumab	27.12	3.35	19	13.67	2.16	19	26.2%	13.45 [11.66, 15.24]
Lemos et al.	27.27	11.87	106	15.31	3.902	106	25.7%	11.96 [9.58, 14.34]
Lim et al.	30.4	8.9	25	17.7	2.5	25	24.0%	12.70 [9.08, 16.32]
Park et al.	37.64	11.68	42	11.48	2.22	42	24.1%	26.16 [22.56, 29.76]
**Total (95% CI)**			**192**			**192**	**100.0%**	**15.95 [10.64, 21.25]**

Heterogeneity: Tau² = 27.08; Chi² = 47.04, df = 3 (P < 0.00001); I² = 94%
Test for overall effect: Z = 5.89 (P < 0.00001)

Mean Difference IV, Random, 95% CI

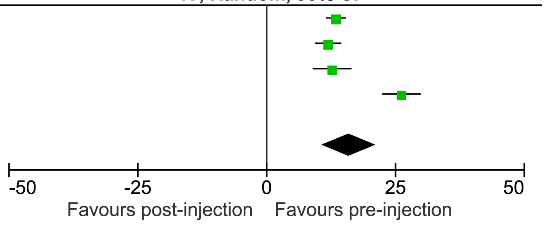

III

Study or Subgroup	post-injection Mean	SD	Total	pre-injection Mean	SD	Total	Weight	Mean Difference IV, Random, 95% CI
Cacciamani et al.	22.76	4.43	25	16.52	3.32	25	27.3%	6.24 [4.07, 8.41]
Kim, GN et al. - bevacizumab	18.48	1.76	19	13.67	2.16	19	40.7%	4.81 [3.56, 6.06]
Lim et al.	20.7	3.9	25	17.7	2.5	25	32.0%	3.00 [1.18, 4.82]
**Total (95% CI)**			**69**			**69**	**100.0%**	**4.62 [3.01, 6.23]**

Heterogeneity: Tau² = 1.25; Chi² = 5.28, df = 2 (P = 0.07); I² = 62%
Test for overall effect: Z = 5.62 (P < 0.00001)

Mean Difference IV, Random, 95% CI

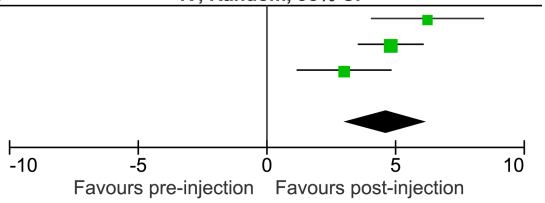

IV

Study or Subgroup	post-injection Mean	SD	Total	pre-injection Mean	SD	Total	Weight	Mean Difference IV, Random, 95% CI
Kim, GN et al. - bevacizumab	15.89	1.27	19	13.67	2.16	19	15.4%	2.22 [1.09, 3.35]
Lee, K et al. - hypertensive group	14.58	2.88	61	13.11	2.31	61	16.0%	1.47 [0.54, 2.40]
Lee, K et al. - nonhypertensive group	13.78	3.97	74	12.77	2.97	74	15.3%	1.01 [-0.12, 2.14]
Lim et al.	17.7	2.8	25	17.7	2.5	25	14.1%	0.00 [-1.47, 1.47]
Mazzula et al.	20.1	4.7	29	15.1	2.35	29	12.4%	5.00 [3.09, 6.91]
Omay et al. - bevacizumab	21.81	7.27	97	16.77	4.47	97	13.2%	5.04 [3.34, 6.74]
Park et al.	14.88	4.77	42	11.48	2.22	42	13.6%	3.40 [1.81, 4.99]
**Total (95% CI)**			**347**			**347**	**100.0%**	**2.48 [1.24, 3.72]**

Heterogeneity: Tau² = 2.26; Chi² = 36.11, df = 6 (P < 0.00001); I² = 83%
Test for overall effect: Z = 3.93 (P < 0.0001)

Mean Difference IV, Random, 95% CI

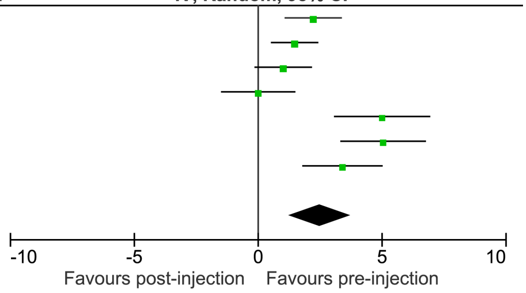

V

Study or Subgroup	post-injection Mean	SD	Total	pre-injection Mean	SD	Total	Weight	Mean Difference IV, Random, 95% CI
Kernt et al.	13.6	3.41	45	13.9	2.6	45	33.9%	-0.30 [-1.55, 0.95]
Kim, GN et al. - bevacizumab	16.77	2.09	19	13.67	2.16	19	33.2%	3.10 [1.75, 4.45]
Lemos et al.	17.59	6.24	106	15.31	3.902	106	32.9%	2.28 [0.88, 3.68]
**Total (95% CI)**			**170**			**170**	**100.0%**	**1.68 [-0.39, 3.75]**

Heterogeneity: Tau² = 2.89; Chi² = 14.47, df = 2 (P = 0.0007); I² = 86%
Test for overall effect: Z = 1.59 (P = 0.11)

Mean Difference IV, Random, 95% CI

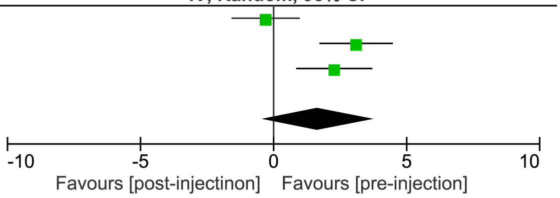

VI

Study or Subgroup	post-injection Mean	SD	Total	pre-injection Mean	SD	Total	Weight	Mean Difference IV, Random, 95% CI
Kernt et al.	12.7	3.1	45	13.9	2.6	45	17.0%	-1.20 [-2.38, -0.02]
Kim, GN et al. - bevacizumab	14.5	2.28	19	13.67	2.16	19	14.8%	0.83 [-0.58, 2.24]
Lee, K et al. - hypertensive group	11.99	2.6	61	13.11	2.31	61	20.4%	-1.12 [-1.99, -0.25]
Lee, K et al. - nonhypertensive group	10.82	2.63	74	12.77	2.97	74	20.0%	-1.95 [-2.85, -1.05]
Omay et al. - bevacizumab	14.32	4.58	97	16.77	4.47	97	16.1%	-2.45 [-3.72, -1.18]
Ozkan et al.	14.8	2.7	21	15.6	3.2	21	11.6%	-0.80 [-2.59, 0.99]
**Total (95% CI)**			**317**			**317**	**100.0%**	**-1.19 [-2.00, -0.37]**

Heterogeneity: Tau² = 0.65; Chi² = 14.35, df = 5 (P = 0.01); I² = 65%
Test for overall effect: Z = 2.86 (P = 0.004)

Mean Difference IV, Random, 95% CI

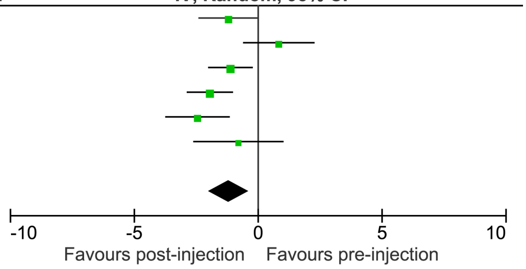

VII

Study or Subgroup	post-injection Mean	post-injection SD	post-injection Total	pre-injection Mean	pre-injection SD	pre-injection Total	Weight	Mean Difference IV, Random, 95% CI
Faghihi et al.	16.4	4.1	64	15.7	2.9	64	12.9%	0.70 [-0.53, 1.93]
Kim, GN et al. - bevacizumab	15.36	1.85	19	13.67	2.16	19	12.7%	1.69 [0.41, 2.97]
Kim, JY et al.	12.5	2.63	22	13.09	2.07	22	12.2%	-0.59 [-1.99, 0.81]
Lee, K et al. - hypertensive group	12.16	2.31	61	13.11	2.31	61	14.7%	-0.95 [-1.77, -0.13]
Lee, K et al. - nonhypertensive group	11.71	2.47	74	12.77	2.97	74	14.4%	-1.06 [-1.94, -0.18]
Omay et al. - bevacizumab	14.49	4.31	97	16.77	4.47	97	12.9%	-2.28 [-3.52, -1.04]
Ozkan et al.	16.4	3.3	21	15.6	3.2	21	9.7%	0.80 [-1.17, 2.77]
Totan et al.	18.4	3.7	30	16.5	3.3	30	10.5%	1.90 [0.13, 3.67]
**Total (95% CI)**			**388**			**388**	**100.0%**	**-0.08 [-1.03, 0.88]**

Heterogeneity: Tau² = 1.46; Chi² = 34.07, df = 7 (P < 0.0001); I² = 79%
Test for overall effect: Z = 0.15 (P = 0.88)

Mean Difference IV, Random, 95% CI

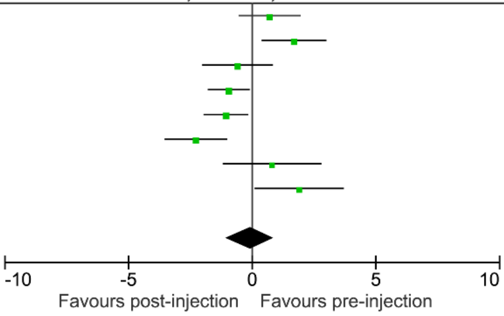

VIII

Study or Subgroup	post-injection Mean	post-injection SD	post-injection Total	pre-injection Mean	pre-injection SD	pre-injection Total	Weight	Mean Difference IV, Random, 95% CI
Ding et al.	13.2	2.87	16	12.2	2	16	17.5%	1.00 [-0.71, 2.71]
Guler et al.	14.29	3.91	43	14.32	4.25	43	17.4%	-0.03 [-1.76, 1.70]
Kernt et al.	12.7	3	45	13.9	2.6	45	21.9%	-1.20 [-2.36, -0.04]
Lemos et al.	16.86	3.62	106	15.31	3.902	106	23.1%	1.55 [0.54, 2.56]
Song, J.H. et al.	15.4	2.75	38	16.1	3.34	38	20.2%	-0.70 [-2.08, 0.68]
**Total (95% CI)**			**248**			**248**	**100.0%**	**0.12 [-1.05, 1.29]**

Heterogeneity: Tau² = 1.28; Chi² = 14.96, df = 4 (P = 0.005); I² = 73%
Test for overall effect: Z = 0.21 (P = 0.84)

Mean Difference IV, Random, 95% CI

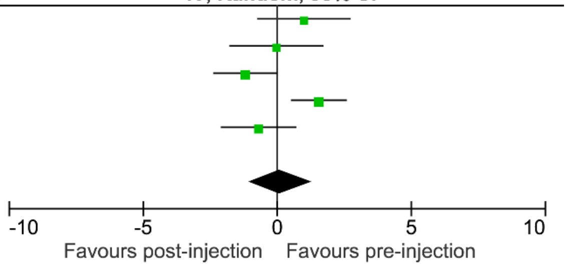

IX

Study or Subgroup	post-injection Mean	post-injection SD	post-injection Total	pre-injection Mean	pre-injection SD	pre-injection Total	Weight	Mean Difference IV, Random, 95% CI
Ding et al.	13.22	2.94	16	12.2	2	16	8.1%	1.02 [-0.72, 2.76]
Gado et al.	17	1.598	30	17	1.839	30	14.0%	0.00 [-0.87, 0.87]
Guler et al.	13.23	4.37	43	14.32	4.25	43	7.7%	-1.09 [-2.91, 0.73]
Kim, GN et al. - bevacizumab	14.86	2.73	19	13.67	2.16	19	9.1%	1.19 [-0.38, 2.76]
Kim, JY et al.	12.23	2.72	22	13.09	2.07	22	10.0%	-0.86 [-2.29, 0.57]
Omay et al. - bevacizumab	15.19	4.68	97	16.77	4.47	97	10.9%	-1.58 [-2.87, -0.29]
Ozkan et al.	16.3	2.6	21	15.6	3.2	21	8.0%	0.70 [-1.06, 2.46]
Rensch et al.	14.5	3.2	25	14.2	3.2	25	8.0%	0.30 [-1.47, 2.07]
Song, J.H. et al.	16	2.86	38	16.1	3.34	38	10.2%	-0.10 [-1.50, 1.30]
Totan et al.	19.5	5.3	30	16.5	3.3	30	6.0%	3.00 [0.77, 5.23]
Tsai et al.	15.3	3	23	14.4	3.1	23	8.0%	0.90 [-0.86, 2.66]
**Total (95% CI)**			**364**			**364**	**100.0%**	**0.17 [-0.49, 0.84]**

Heterogeneity: Tau² = 0.62; Chi² = 20.69, df = 10 (P = 0.02); I² = 52%
Test for overall effect: Z = 0.50 (P = 0.61)

Mean Difference IV, Random, 95% CI

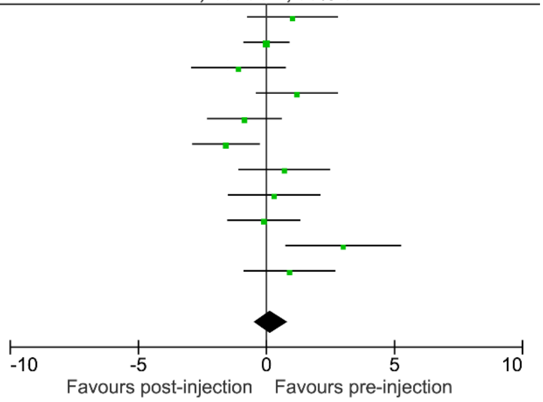

X

Study or Subgroup	post-injection Mean	post-injection SD	post-injection Total	pre-injection Mean	pre-injection SD	pre-injection Total	Weight	Mean Difference IV, Fixed, 95% CI
Gado et al.	17	1.598	30	17	1.839	30	26.3%	0.00 [-0.87, 0.87]
Kim, JY et al.	12.55	2.09	22	13.09	2.07	22	13.2%	-0.54 [-1.77, 0.69]
Lee, K et al. - hypertensive group	12.75	2.51	61	13.11	2.31	61	27.2%	-0.36 [-1.22, 0.50]
Lee, K et al. - nonhypertensive group	12.55	3.09	74	12.77	2.97	74	20.9%	-0.22 [-1.20, 0.76]
Song, J.H. et al.	16.4	2.2	38	16.1	3.34	38	12.3%	0.30 [-0.97, 1.57]
**Total (95% CI)**			**225**			**225**	**100.0%**	**-0.18 [-0.63, 0.27]**

Heterogeneity: Chi² = 1.22, df = 4 (P = 0.88); I² = 0%
Test for overall effect: Z = 0.78 (P = 0.43)

Mean Difference IV, Fixed, 95% CI

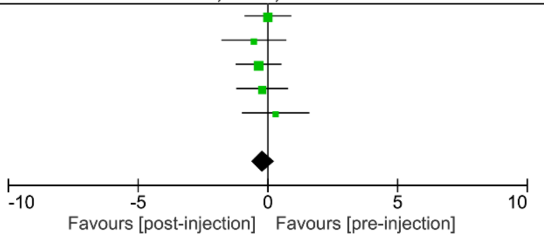

XI

Study or Subgroup	post-injection Mean	post-injection SD	post-injection Total	pre-injection Mean	pre-injection SD	pre-injection Total	Weight	Mean Difference IV, Fixed, 95% CI
Ding et al.	13.62	2.91	16	12.2	2	16	3.2%	1.42 [-0.31, 3.15]
Gado et al.	16	1.343	30	17	1.839	30	14.4%	-1.00 [-1.81, -0.19]
Kernt et al.	14.7	2.9	45	13.9	2.6	45	7.4%	0.80 [-0.34, 1.94]
Kim, JY et al.	12	2.78	22	13.09	2.07	22	4.6%	-1.09 [-2.54, 0.36]
Lee, K et al. - hypertensive group	13.1	2.4	61	13.11	2.31	61	13.7%	-0.01 [-0.85, 0.83]
Lee, K et al. - nonhypertensive group	12.79	3.4	74	12.77	2.97	74	9.0%	0.02 [-1.01, 1.05]
Maray et al.	15.13	2.21	30	15.47	2.93	30	5.5%	-0.34 [-1.65, 0.97]
Ozkan et al.	15.3	2.8	21	15.6	3.2	21	2.9%	-0.30 [-2.12, 1.52]
Ramezani et al.	12.8	2	43	13.3	2.7	43	9.5%	-0.50 [-1.50, 0.50]
Rensch et al.	14.2	2.8	25	14.2	3.2	25	3.4%	0.00 [-1.67, 1.67]
Soheilian et al.	14.7	1.6	44	14.2	2	44	16.7%	0.50 [-0.26, 1.26]
Tas et al.	13.72	3.04	40	13.31	3.22	40	5.1%	0.41 [-0.96, 1.78]
Totan et al.	18.5	4.8	30	16.5	3.3	30	2.2%	2.00 [-0.08, 4.08]
Tsai et al.	14.7	3.6	23	14.4	3.1	23	2.5%	0.30 [-1.64, 2.24]
**Total (95% CI)**			**504**			**504**	**100.0%**	**-0.01 [-0.32, 0.30]**

Heterogeneity: Chi² = 19.41, df = 13 (P = 0.11); I² = 33%
Test for overall effect: Z = 0.05 (P = 0.96)

Mean Difference IV, Fixed, 95% CI

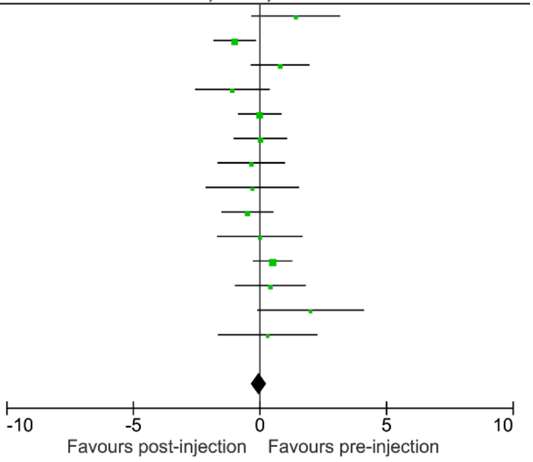

XII

Study or Subgroup	post-injection Mean	SD	Total	pre-injection Mean	SD	Total	Weight	Mean Difference IV, Fixed, 95% CI
Gado et al.	16	1.623	30	17	1.839	30	27.3%	-1.00 [-1.88, -0.12]
Lee, K et al. - hypertensive group	13.22	2.71	61	13.11	2.31	61	26.4%	0.11 [-0.78, 1.00]
Lee, K et al. - nonhypertensive group	12.78	2.58	74	12.77	2.97	74	26.2%	0.01 [-0.89, 0.91]
Ramezani et al.	13.3	2.1	43	13.3	2.7	43	20.1%	0.00 [-1.02, 1.02]
**Total (95% CI)**			**208**			**208**	**100.0%**	**-0.24 [-0.70, 0.22]**

Heterogeneity: Chi² = 3.98, df = 3 (P = 0.26); I² = 25%
Test for overall effect: Z = 1.03 (P = 0.30)

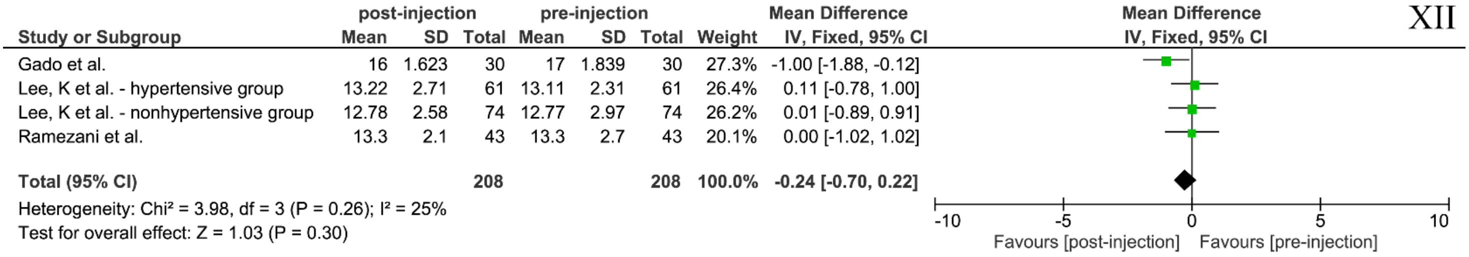

XIII

Study or Subgroup	post-injection Mean	SD	Total	pre-injection Mean	SD	Total	Weight	Mean Difference IV, Fixed, 95% CI
Ding et al.	13.84	3.08	16	12.2	2	16	3.2%	1.64 [-0.16, 3.44]
Gado et al.	16	2.024	30	17	1.839	30	10.9%	-1.00 [-1.98, -0.02]
Jaffar et al.	14.21	3.12	100	13.8	3.16	100	13.8%	0.41 [-0.46, 1.28]
Kim, D et al.	13.61	3.05	83	14.11	2.76	83	13.4%	-0.50 [-1.38, 0.38]
Kim, JY et al.	12.32	2.92	22	13.09	2.07	22	4.7%	-0.77 [-2.27, 0.73]
Lee, K et al. - hypertensive group	13.2	2.01	61	13.11	2.31	61	17.7%	0.09 [-0.68, 0.86]
Lee, K et al. - nonhypertensive group	13.06	2.26	74	12.77	2.97	74	14.5%	0.29 [-0.56, 1.14]
Ozkan et al.	15.8	2.8	21	15.6	3.2	21	3.2%	0.20 [-1.62, 2.02]
Ramezani et al.	12.3	2.3	43	13.3	2.7	43	9.3%	-1.00 [-2.06, 0.06]
Rensch et al.	14	3.1	25	14.2	3.2	25	3.4%	-0.20 [-1.95, 1.55]
Totan et al.	17.1	4.1	30	16.5	3.3	30	2.9%	0.60 [-1.28, 2.48]
Tsai et al.	14	3.4	23	14.4	3.1	23	3.0%	-0.40 [-2.28, 1.48]
**Total (95% CI)**			**528**			**528**	**100.0%**	**-0.13 [-0.46, 0.19]**

Heterogeneity: Chi² = 14.23, df = 11 (P = 0.22); I² = 23%
Test for overall effect: Z = 0.80 (P = 0.42)

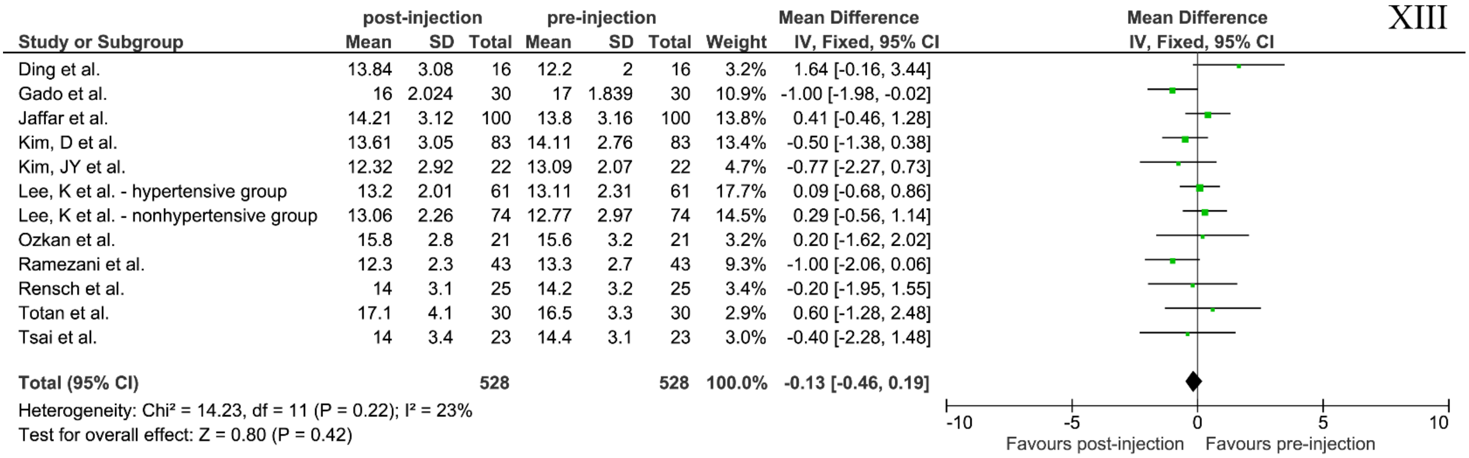

IXV

Study or Subgroup	post-injection Mean	SD	Total	pre-injection Mean	SD	Total	Weight	Mean Difference IV, Fixed, 95% CI
Kim, D et al.	13.74	3.21	83	14.11	2.76	83	41.6%	-0.37 [-1.28, 0.54]
Jaffar et al.	13.79	3.07	100	13.8	3.16	100	46.3%	-0.01 [-0.87, 0.85]
Higashiyama et al.	13.9	2.7	22	13.7	3	22	12.1%	0.20 [-1.49, 1.89]
**Total (95% CI)**			**205**			**205**	**100.0%**	**-0.13 [-0.72, 0.45]**

Heterogeneity: Chi² = 0.49, df = 2 (P = 0.78); I² = 0%
Test for overall effect: Z = 0.45 (P = 0.65)

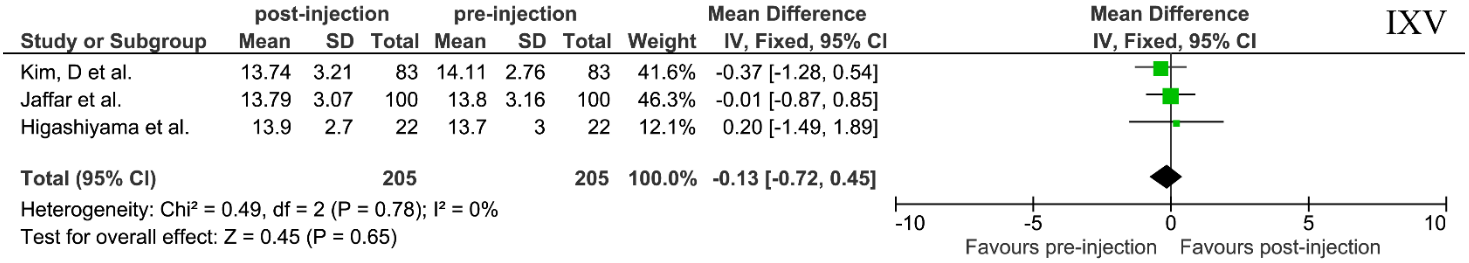

Black diamonds indicate the overall weighted mean difference (WMD). The size of the green box is inversely proportional to the variance. Horizontal lines indicate 95% CI. The vertical line in each panel shows the value for no effect (WMD = 0).

IOP = intra-ocular pressure; CI = confidence interval; SD = standard deviation; I = 1 minute or less after injection; II = 5 minutes; III = 15 minutes; IV = 30 minutes; V = 1 hour; VI = 1 day; VII = 1 week; VIII = 2 weeks; IX = 1 month; X = 2 months; XI = 3 months; XII = 4 months; XIII = 6 months; IXV = 12 months.

FIGURE S3. Weighted mean difference in intraocular pressure during follow-up after ranibizumab intravitreal injection

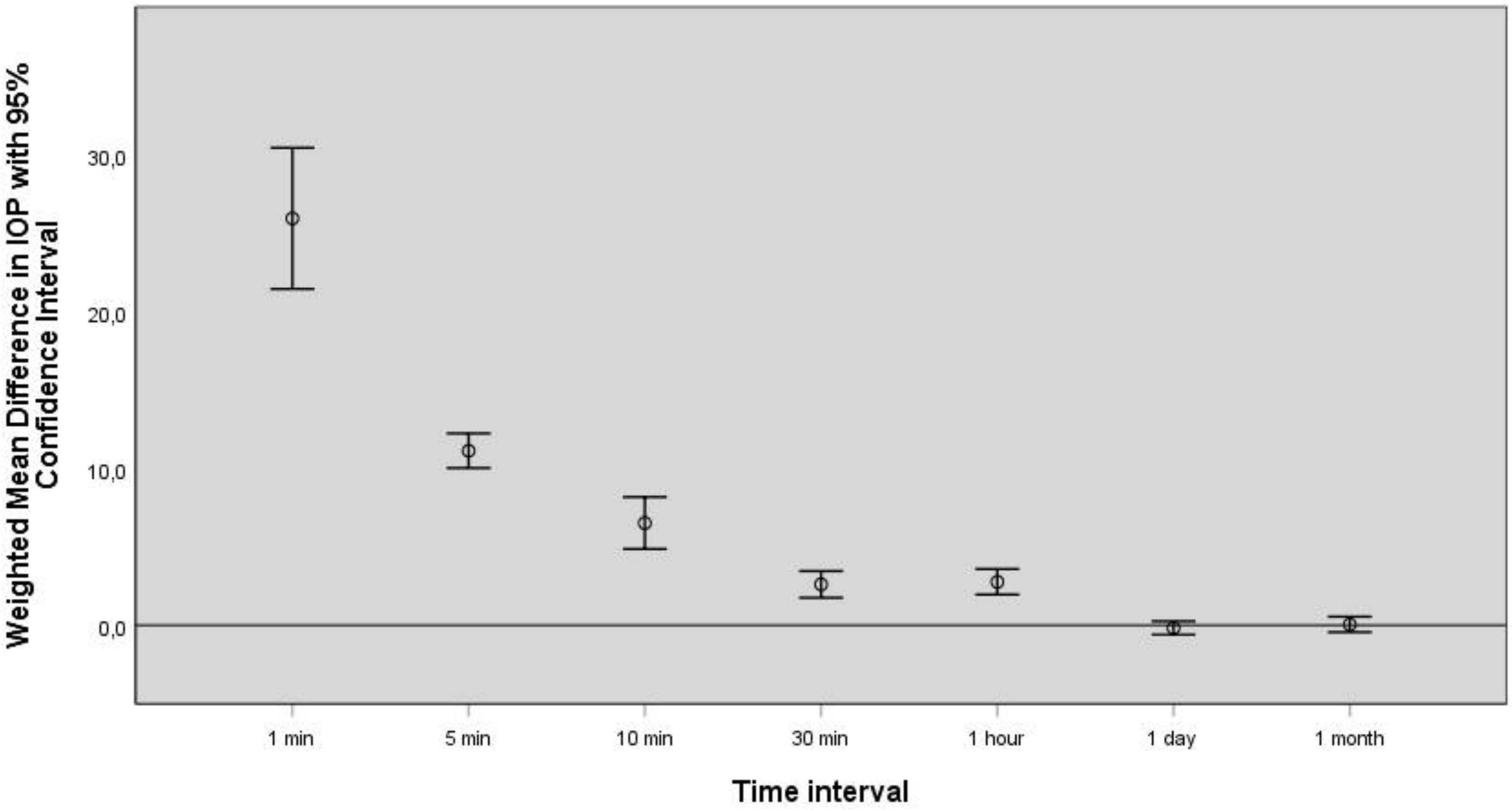

IOP = intraocular pressure

FIGURE S4. Meta-analyses for the association of ranibizumab and IOP.

I

Study or Subgroup	post-injection Mean	SD	Total	pre-injection Mean	SD	Total	Weight	Mean Difference IV, Random, 95% CI
Arikan et al.	28	14.6	106	14.9	2.4	106	13.6%	13.10 [10.28, 15.92]
Fuest et al.	47.2	11.2	29	14.3	0.6	29	12.8%	32.90 [28.82, 36.98]
Gismondi et al.	44.1	10.6	54	16.3	3	54	13.5%	27.80 [24.86, 30.74]
Gregori et al. - cotton swabs group	41.2	9.9	48	15.5	3.3	48	13.5%	25.70 [22.75, 28.65]
Gregori et al. - gel group	46.8	10.5	48	15.9	3	48	13.4%	30.90 [27.81, 33.99]
Hohn et al.	43.4	15.6	31	15.14	2.95	31	11.7%	28.26 [22.67, 33.85]
Karakurt et al. - ranibizumab	37.2	6.41	81	15.14	2.95	81	14.1%	22.06 [20.52, 23.60]
Murray et al.	44.5	19.8	12	15.1	5.5	12	7.3%	29.40 [17.77, 41.03]
**Total (95% CI)**			**409**			**409**	**100.0%**	**25.95 [21.46, 30.45]**

Heterogeneity: Tau² = 36.70; Chi² = 111.77, df = 7 (P < 0.00001); I² = 94%
Test for overall effect: Z = 11.31 (P < 0.00001)

Mean Difference IV, Random, 95% CI

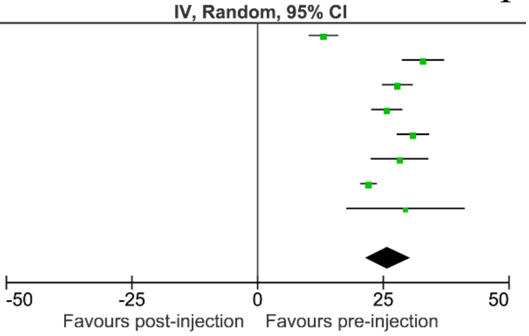

II

Study or Subgroup	post-injection Mean	SD	Total	pre-injection Mean	SD	Total	Weight	Mean Difference IV, Fixed, 95% CI
Fuest et al.	24.5	5.8	29	14.3	0.6	29	27.1%	10.20 [8.08, 12.32]
Gismondi et al.	29	9.6	54	16.3	3	54	17.0%	12.70 [10.02, 15.38]
Goktas et al.	24.8	9.5	31	14.5	2.3	31	10.3%	10.30 [6.86, 13.74]
Kim, GN et al. - ranibizumab	25.19	1.04	10	14.08	2.48	10	44.0%	11.11 [9.44, 12.78]
Murray et al.	31.4	14.4	12	15.1	5.5	12	1.6%	16.30 [7.58, 25.02]
**Total (95% CI)**			**136**			**136**	**100.0%**	**11.13 [10.03, 12.24]**

Heterogeneity: Chi² = 3.63, df = 4 (P = 0.46); I² = 0%
Test for overall effect: Z = 19.74 (P < 0.00001)

Mean Difference IV, Fixed, 95% CI

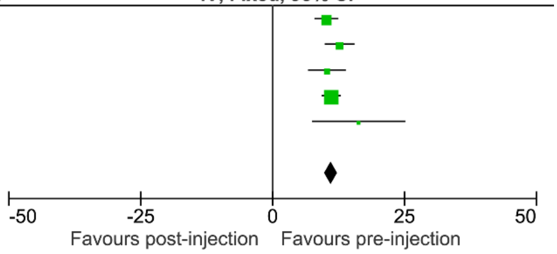

III

Study or Subgroup	post-injection Mean	SD	Total	pre-injection Mean	SD	Total	Weight	Mean Difference IV, Random, 95% CI
Fuest et al.	20.6	4.4	29	14.3	0.6	29	17.4%	6.30 [4.68, 7.92]
Gismondi et al.	25.8	7.9	54	16.3	3	54	15.1%	9.50 [7.25, 11.75]
Hohn et al.	23.1	8.2	31	22.5	5.9	31	10.6%	0.60 [-2.96, 4.16]
Karakurt et al. - ranibizumab	23.62	5.77	81	15.14	2.95	81	18.1%	8.48 [7.07, 9.89]
Kim, GN et al. - ranibizumab	20.22	2.73	10	14.08	2.48	10	14.9%	6.14 [3.85, 8.43]
Murray et al.	24.5	11.7	12	15.1	5.5	12	4.1%	9.40 [2.09, 16.71]
Song, S. et al.	21.35	5.88	203	15.82	2.57	203	19.7%	5.53 [4.65, 6.41]
**Total (95% CI)**			**420**			**420**	**100.0%**	**6.52 [4.87, 8.18]**

Heterogeneity: Tau² = 3.41; Chi² = 30.27, df = 6 (P < 0.0001); I² = 80%
Test for overall effect: Z = 7.73 (P < 0.00001)

Mean Difference IV, Random, 95% CI

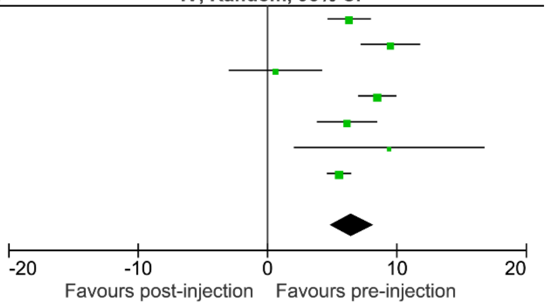

IV

Study or Subgroup	post-injection Mean	SD	Total	pre-injection Mean	SD	Total	Weight	Mean Difference IV, Random, 95% CI
Demirel et al.	18.5	4.32	202	15.11	2.8	202	14.1%	3.39 [2.68, 4.10]
Gismondi et al.	21.9	5.6	54	16.3	3	54	9.7%	5.60 [3.91, 7.29]
Goktas et al.	17.3	4.1	31	14.5	2.3	31	9.8%	2.80 [1.15, 4.45]
Kim, GN et al. - ranibizumab	15.98	2.71	10	14.08	2.48	10	7.4%	1.90 [-0.38, 4.18]
Murray et al.	20.6	9.5	12	15.1	5.5	12	1.7%	5.50 [-0.71, 11.71]
Omay et al. - ranibizumab	23.67	10.12	12	14.08	2.77	12	1.8%	9.59 [3.65, 15.53]
Sengul et al. - 10/20 injections	17.35	1.8	62	16.04	2.07	62	14.2%	1.31 [0.63, 1.99]
Sengul et al. - 20+ injections	17.5	2.07	44	15.79	2.32	44	13.2%	1.71 [0.79, 2.63]
Sengul et al. - 3/10 injections	17.24	2.22	62	16.05	2.13	62	13.9%	1.19 [0.42, 1.96]
Song, S. et al.	18.17	4.06	203	15.82	2.57	203	14.3%	2.35 [1.69, 3.01]
**Total (95% CI)**			**692**			**692**	**100.0%**	**2.61 [1.76, 3.46]**

Heterogeneity: Tau² = 1.21; Chi² = 48.36, df = 9 (P < 0.00001); I² = 81%
Test for overall effect: Z = 6.01 (P < 0.00001)

Mean Difference IV, Random, 95% CI

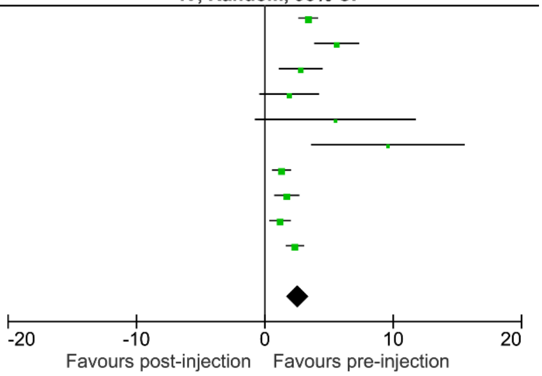

V

Study or Subgroup	post-injection Mean	SD	Total	pre-injection Mean	SD	Total	Weight	Mean Difference IV, Fixed, 95% CI
Gismondi et al.	18.8	6.1	54	16.3	3	54	20.2%	2.50 [0.69, 4.31]
Karakurt et al. - ranibizumab	18.22	3.67	81	15.14	2.95	81	63.1%	3.08 [2.05, 4.11]
Kim, GN et al. - ranibizumab	16.02	2.04	10	14.08	2.48	10	16.7%	1.94 [-0.05, 3.93]
**Total (95% CI)**			**145**			**145**	**100.0%**	**2.77 [1.96, 3.59]**

Heterogeneity: Chi² = 1.10, df = 2 (P = 0.58); I² = 0%
Test for overall effect: Z = 6.67 (P < 0.00001)

Mean Difference IV, Fixed, 95% CI

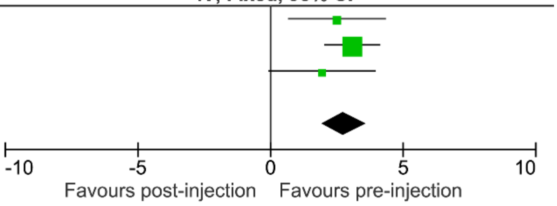

VI

Study or Subgroup	post-injection Mean	post-injection SD	post-injection Total	pre-injection Mean	pre-injection SD	pre-injection Total	Weight	Mean Difference IV, Random, 95% CI
Demirel et al.	14.21	2.9	202	15.11	2.8	202	15.7%	-0.90 [-1.46, -0.34]
Gismondi et al.	16.1	3	54	16.3	3	54	8.4%	-0.20 [-1.33, 0.93]
Goktas et al.	13.3	2.4	31	14.5	2.3	31	8.1%	-1.20 [-2.37, -0.03]
Karakurt et al. - ranibizumab	15.83	3.2	81	15.14	2.95	81	10.3%	0.69 [-0.26, 1.64]
Kim, GN et al. - ranibizumab	14.21	2.83	10	14.08	2.48	10	2.8%	0.13 [-2.20, 2.46]
Omay et al. - ranibizumab	12.25	2.13	12	14.08	2.77	12	3.8%	-1.83 [-3.81, 0.15]
Sengul et al. - 10/20 injections	16.35	2.38	62	16.04	2.07	62	12.3%	0.31 [-0.48, 1.10]
Sengul et al. - 20+ injections	15.95	2.48	44	15.79	2.32	44	9.7%	0.16 [-0.84, 1.16]
Sengul et al. - 3/10 injections	16.26	2.11	62	16.05	2.13	62	12.9%	0.21 [-0.54, 0.96]
Song, S. et al.	15.84	2.92	203	15.82	2.57	203	16.0%	0.02 [-0.52, 0.56]
**Total (95% CI)**			**761**			**761**	**100.0%**	**-0.17 [-0.59, 0.26]**

Heterogeneity: Tau² = 0.21; Chi² = 18.87, df = 9 (P = 0.03); I² = 52%

Test for overall effect: Z = 0.77 (P = 0.44)

Mean Difference IV, Random, 95% CI

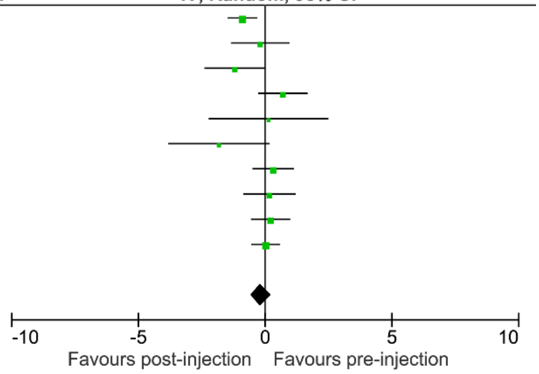

VII

Study or Subgroup	post-injection Mean	post-injection SD	post-injection Total	pre-injection Mean	pre-injection SD	pre-injection Total	Weight	Mean Difference IV, Fixed, 95% CI
Kaldirim et al. - ranibizumab	16.13	0.99	22	16.09	1.27	22	53.6%	0.04 [-0.63, 0.71]
Karakurt et al. - ranibizumab	15.23	2.16	81	15.14	2.95	81	38.3%	0.09 [-0.71, 0.89]
Kim, GN et al. - ranibizumab	14.08	2.48	10	14.08	2.48	10	5.1%	0.00 [-2.17, 2.17]
Omay et al. - ranibizumab	14	4.15	12	14.08	2.77	12	3.0%	-0.08 [-2.90, 2.74]
**Total (95% CI)**			**125**			**125**	**100.0%**	**0.05 [-0.44, 0.55]**

Heterogeneity: Chi² = 0.02, df = 3 (P = 1.00); I² = 0%

Test for overall effect: Z = 0.21 (P = 0.83)

Mean Difference IV, Fixed, 95% CI

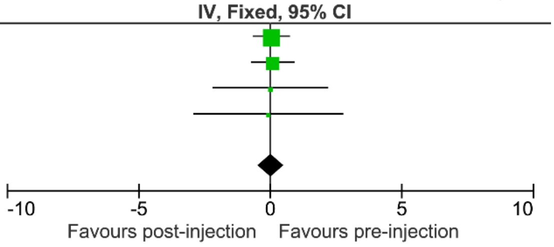

Black diamonds indicate the overall weighted mean difference (WMD). The size of the green box is inversely proportional to the variance. Horizontal lines indicate 95% CI. The vertical line in each panel shows the value for no effect (WMD = 0).

IOP = intraocular pressure; CI = confidence interval; SD = standard deviation; I = 1 minute after injection; II = 5 minutes; III = 10 minutes; IV = 30 minutes; V = 1 hour; VI = 1 day; VII = 1 month.

Figure S5. Meta-analyses for the association of anti-VEGF and IOP.

I

Study or Subgroup	post-injection Mean	SD	Total	pre-injection Mean	SD	Total	Weight	Mean Difference IV, Random, 95% CI
Arikan et al.	28	14.6	106	14.9	2.4	106	6.8%	13.10 [10.28, 15.92]
Cacciamani et al.	38.44	7.92	25	16.52	3.32	25	6.7%	21.92 [18.55, 25.29]
el-Chehab et al.	42.7	3	30	12.9	1.3	30	6.9%	29.80 [28.63, 30.97]
Fuest et al.	47.2	11.2	29	14.3	0.6	29	6.7%	32.90 [28.82, 36.98]
Gismondi et al.	44.1	10.6	54	16.3	3	54	6.8%	27.80 [24.86, 30.74]
Gregori et al. - cotton swabs group	46.8	10.5	48	15.9	3	48	6.8%	30.90 [27.81, 33.99]
Gregori et al. - gel group	41.2	9.9	48	15.5	3.3	48	6.8%	25.70 [22.75, 28.65]
Hohn et al.	43.4	15.6	31	22.5	5.9	31	6.4%	20.90 [15.03, 26.77]
Karakurt et al. - aflibercept	36.75	5.98	53	15.64	3.18	53	6.9%	21.11 [19.29, 22.93]
Karakurt et al. - ranibizumab	37.2	6.41	81	15.14	2.95	81	6.9%	22.06 [20.52, 23.60]
Kernt et al.	14.5	4.4	45	13.9	2.6	45	6.9%	0.60 [-0.89, 2.09]
Lee, JW et al.	43.81	9.69	65	16.66	3.5	65	6.8%	27.15 [24.65, 29.65]
Lemos-Reiss et al.	42.1	14.5	291	18	5.9	291	6.9%	24.10 [22.30, 25.90]
Lim et al.	43.1	9.4	25	17.7	2.5	25	6.7%	25.40 [21.59, 29.21]
Murray et al.	44.5	19.8	12	15.1	5.5	12	5.2%	29.40 [17.77, 41.03]
**Total (95% CI)**			**943**			**943**	**100.0%**	**23.41 [18.12, 28.70]**

Heterogeneity: Tau² = 105.18; Chi² = 1104.57, df = 14 (P < 0.00001); I² = 99%
Test for overall effect: Z = 8.67 (P < 0.00001)

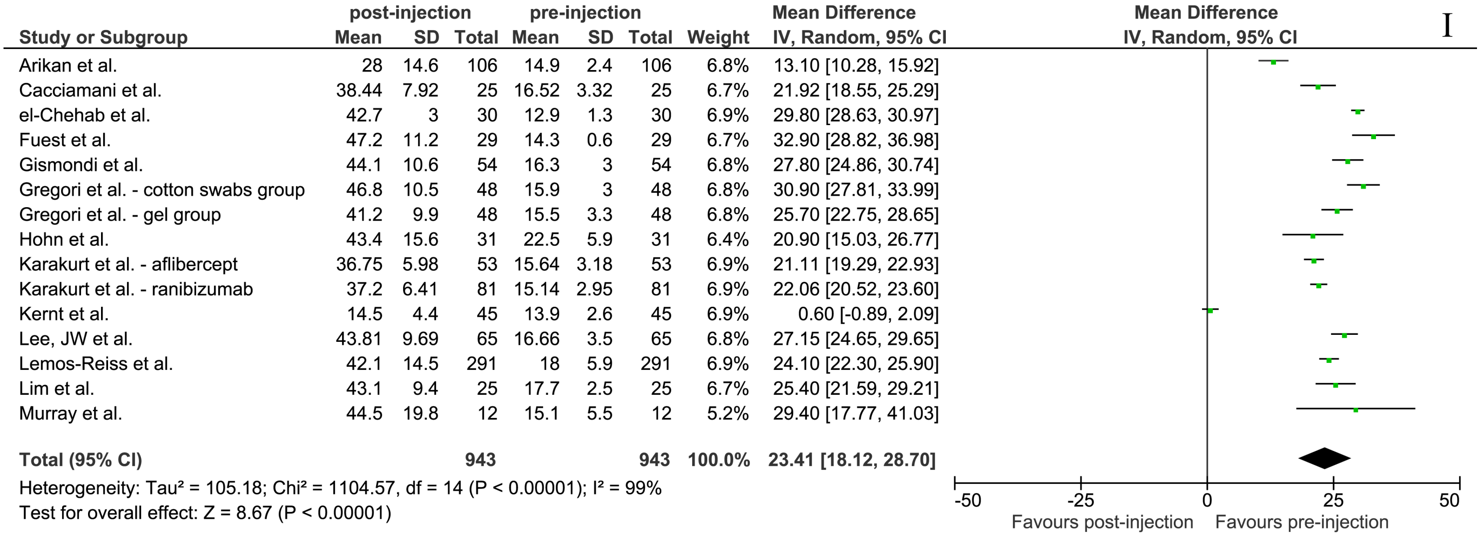

II

Study or Subgroup	post-injection Mean	SD	Total	pre-injection Mean	SD	Total	Weight	Mean Difference IV, Random, 95% CI
el-Chehab et al.	23.6	3.1	30	12.9	1.3	30	11.1%	10.70 [9.50, 11.90]
Fuest et al.	24.5	5.8	29	14.3	0.6	29	10.2%	10.20 [8.08, 12.32]
Gismondi et al.	29	9.6	54	16.3	3	54	9.5%	12.70 [10.02, 15.38]
Goktas et al.	24.8	9.5	31	14.5	2.3	31	8.5%	10.30 [6.86, 13.74]
Kim, GN et al. - bevacizumab	27.12	3.35	19	13.67	2.16	19	10.5%	13.45 [11.66, 15.24]
Kim, GN et al. - ranibizumab	25.19	1.04	10	14.08	2.48	10	10.6%	11.11 [9.44, 12.78]
Lemos et al.	27.27	11.87	106	15.31	3.902	106	9.8%	11.96 [9.58, 14.34]
Lim et al.	30.4	8.9	25	17.7	2.5	25	8.2%	12.70 [9.08, 16.32]
Murray et al.	31.4	14.4	12	15.1	5.5	12	3.5%	16.30 [7.58, 25.02]
Park et al.	37.64	11.68	42	11.48	2.22	42	8.3%	26.16 [22.56, 29.76]
Rebodella et al.	25.4	5.8	30	15.1	3.4	30	9.8%	10.30 [7.89, 12.71]
**Total (95% CI)**			**388**			**388**	**100.0%**	**12.86 [10.91, 14.81]**

Heterogeneity: Tau² = 8.58; Chi² = 74.47, df = 10 (P < 0.00001); I² = 87%
Test for overall effect: Z = 12.93 (P < 0.00001)

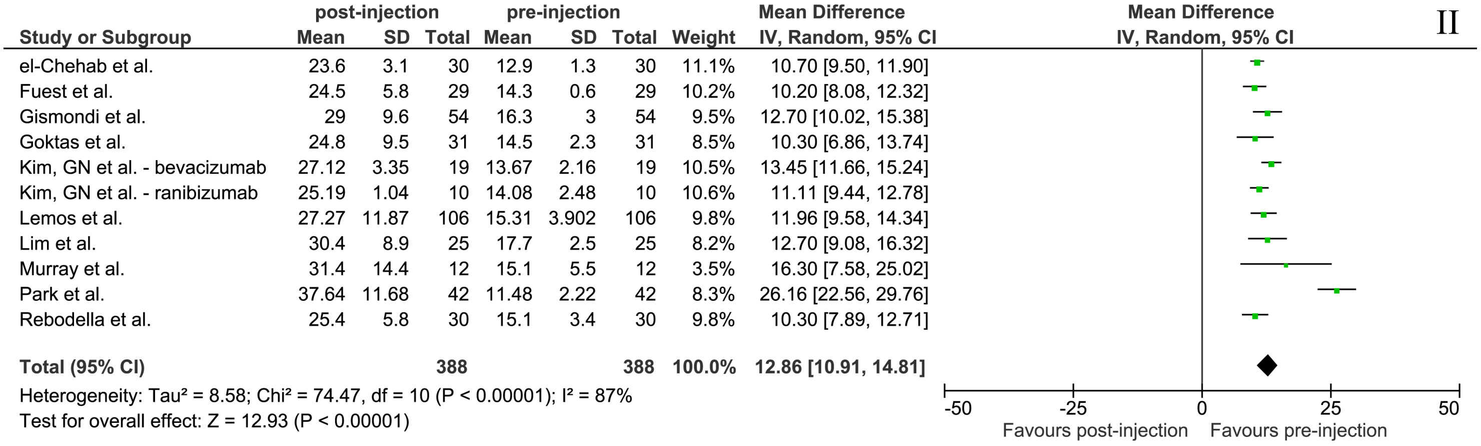

III

Study or Subgroup	post-injection Mean	SD	Total	pre-injection Mean	SD	Total	Weight	Mean Difference IV, Random, 95% CI
Falkenstein et al.	24.56	5.9	70	15.17	3.42	70	11.2%	9.39 [7.79, 10.99]
Fuest et al.	20.6	4.4	29	14.3	0.6	29	11.1%	6.30 [4.68, 7.92]
Gismondi et al.	25.8	7.9	54	16.3	3	54	10.1%	9.50 [7.25, 11.75]
Hohn et al.	23.1	8.2	31	22.5	5.9	31	7.7%	0.60 [-2.96, 4.16]
Karakurt et al. - aflibercept	22.96	5.46	53	15.64	3.18	53	11.0%	7.32 [5.62, 9.02]
Karakurt et al. - ranibizumab	23.62	5.77	81	15.14	2.95	81	11.5%	8.48 [7.07, 9.89]
Kim, GN et al. - bevacizumab	24.34	1.13	19	13.67	2.16	19	11.9%	10.67 [9.57, 11.77]
Kim, GN et al. - ranibizumab	20.22	2.73	10	14.08	2.48	10	10.0%	6.14 [3.85, 8.43]
Murray et al.	24.5	11.7	12	15.1	5.5	12	3.5%	9.40 [2.09, 16.71]
Song, S. et al.	21.34	5.88	203	15.82	2.57	203	12.1%	5.52 [4.64, 6.40]
**Total (95% CI)**			**562**			**562**	**100.0%**	**7.40 [5.81, 9.00]**

Heterogeneity: Tau² = 5.28; Chi² = 79.78, df = 9 (P < 0.00001); I² = 89%
Test for overall effect: Z = 9.09 (P < 0.00001)

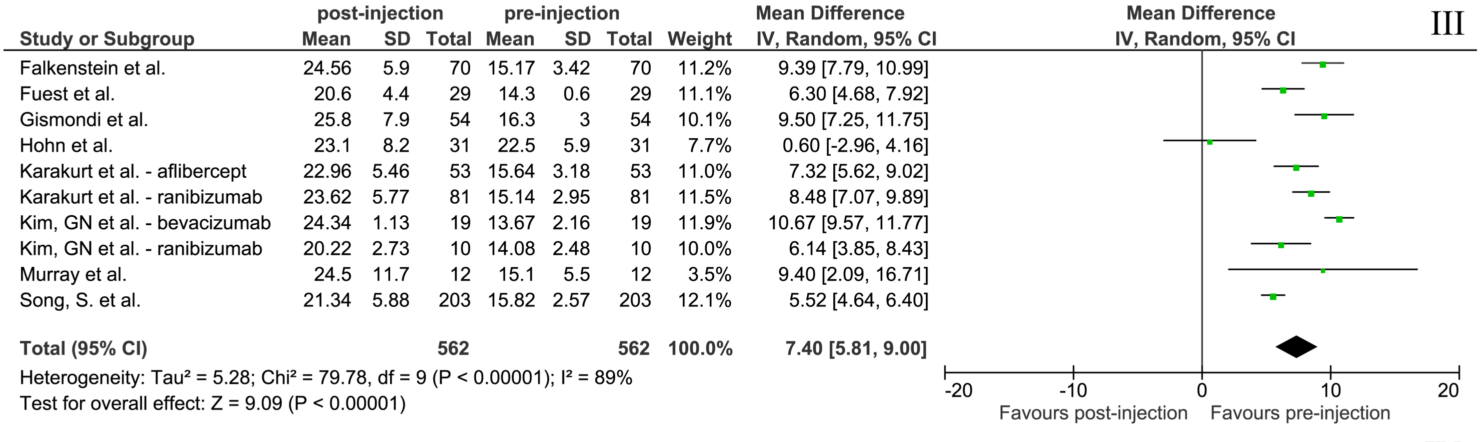

IV

Study or Subgroup	post-injection Mean	SD	Total	pre-injection Mean	SD	Total	Weight	Mean Difference IV, Random, 95% CI
Cacciamani et al.	22.76	4.43	25	16.52	3.32	25	14.7%	6.24 [4.07, 8.41]
el-Chehab et al.	16.2	2.1	30	12.9	1.3	30	19.7%	3.30 [2.42, 4.18]
Gismondi et al.	23.7	6.6	54	16.3	3	54	15.7%	7.40 [5.47, 9.33]
Kim, GN et al. - bevacizumab	18.48	1.76	19	13.67	2.16	19	18.5%	4.81 [3.56, 6.06]
Kim, GN et al. - ranibizumab	21.43	2.24	10	14.08	2.48	10	15.1%	7.35 [5.28, 9.42]
Lim et al.	20.7	3.9	25	17.7	2.5	25	16.2%	3.00 [1.18, 4.82]
**Total (95% CI)**			**163**			**163**	**100.0%**	**5.22 [3.71, 6.74]**

Heterogeneity: Tau² = 2.83; Chi² = 28.32, df = 5 (P < 0.0001); I² = 82%
Test for overall effect: Z = 6.75 (P < 0.00001)

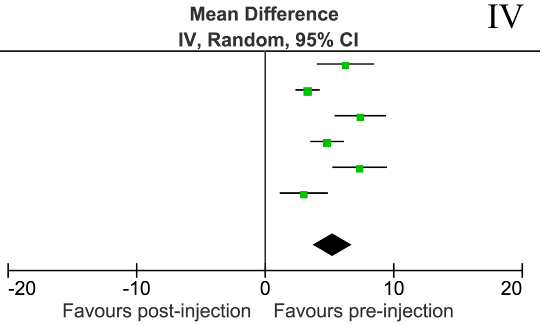

V

Study or Subgroup	post-injection Mean	SD	Total	pre-injection Mean	SD	Total	Weight	Mean Difference IV, Random, 95% CI
Demirel et al.	18.5	4.32	202	15.11	2.8	202	7.1%	3.39 [2.68, 4.10]
Gismondi et al.	21.9	5.6	54	16.3	3	54	5.0%	5.60 [3.91, 7.29]
Goktas et al.	17.3	4.1	31	14.5	2.3	31	5.1%	2.80 [1.15, 4.45]
Kim, GN et al. - bevacizumab	15.89	1.27	19	13.67	2.16	19	6.2%	2.22 [1.09, 3.35]
Kim, GN et al. - ranibizumab	15.98	2.71	10	14.08	2.48	10	3.9%	1.90 [-0.38, 4.18]
Lee, JW et al.	17.57	4.44	65	16.66	3.5	65	5.7%	0.91 [-0.46, 2.28]
Lee, K et al. - hypertensive group	14.58	2.88	61	13.11	2.31	61	6.7%	1.47 [0.54, 2.40]
Lee, K et al. - nonhypertensive group	13.78	3.97	74	12.77	2.97	74	6.2%	1.01 [-0.12, 2.14]
Lim et al.	17.7	2.8	25	17.7	2.5	25	5.5%	0.00 [-1.47, 1.47]
Mazzula et al.	20.1	4.7	29	15.1	2.35	29	4.6%	5.00 [3.09, 6.91]
Murray et al.	20.6	9.5	12	15.1	5.5	12	0.9%	5.50 [-0.71, 11.71]
Omay et al. - bevacizumab	21.81	7.27	97	16.77	4.47	97	5.0%	5.04 [3.34, 6.74]
Omay et al. - ranibizumab	23.67	10.12	12	14.08	2.77	12	1.0%	9.59 [3.65, 15.53]
Park et al.	14.88	4.77	42	11.48	2.22	42	5.2%	3.40 [1.81, 4.99]
Rebodella et al.	19.1	5.4	30	15.1	3.4	30	3.9%	4.00 [1.72, 6.28]
Sengul et al. - 10/20 injections	17.35	1.8	62	16.04	2.07	62	7.1%	1.31 [0.63, 1.99]
Sengul et al. - 20+ injections	17.5	2.07	44	15.79	2.32	44	6.7%	1.71 [0.79, 2.63]
Sengul et al. - 3/10 injections	17.24	2.22	62	16.05	2.13	62	7.0%	1.19 [0.42, 1.96]
Song, S. et al.	18.17	4.06	203	15.82	2.57	203	7.2%	2.35 [1.69, 3.01]
**Total (95% CI)**			**1134**			**1134**	**100.0%**	**2.51 [1.87, 3.15]**

Heterogeneity: Tau² = 1.37; Chi² = 90.39, df = 18 (P < 0.00001); I² = 80%
Test for overall effect: Z = 7.70 (P < 0.00001)

Mean Difference IV, Random, 95% CI

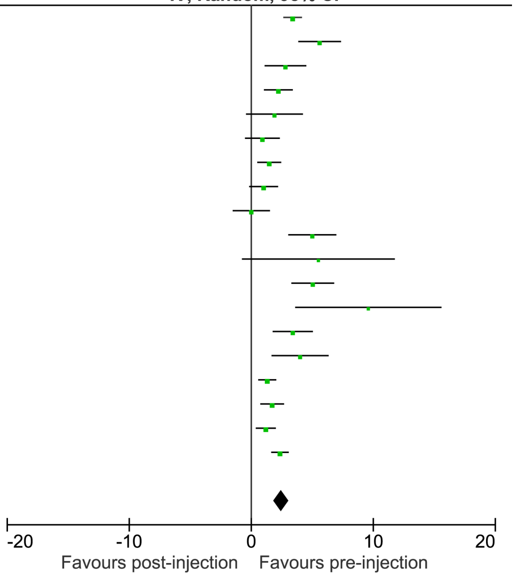

VI

Study or Subgroup	post-injection Mean	SD	Total	pre-injection Mean	SD	Total	Weight	Mean Difference IV, Random, 95% CI
Gismondi et al.	18.8	6.1	54	16.3	3	54	12.2%	2.50 [0.69, 4.31]
Karakurt et al. - aflibercept	17.92	4.31	53	15.64	3.18	53	14.4%	2.28 [0.84, 3.72]
Karakurt et al. - ranibizumab	18.22	3.67	81	15.14	2.95	81	17.0%	3.08 [2.05, 4.11]
Kernt et al.	13.6	3.41	45	13.9	2.6	45	15.6%	-0.30 [-1.55, 0.95]
Kim, GN et al. - bevacizumab	16.77	2.09	19	13.67	2.16	19	15.0%	3.10 [1.75, 4.45]
Kim, GN et al. - ranibizumab	16.02	2.04	10	14.08	2.48	10	11.2%	1.94 [-0.05, 3.93]
Lemos et al.	17.59	6.24	106	15.31	3.902	106	14.6%	2.28 [0.88, 3.68]
**Total (95% CI)**			**368**			**368**	**100.0%**	**2.13 [1.15, 3.10]**

Heterogeneity: Tau² = 1.19; Chi² = 20.00, df = 6 (P = 0.003); I² = 70%
Test for overall effect: Z = 4.26 (P < 0.0001)

Mean Difference IV, Random, 95% CI

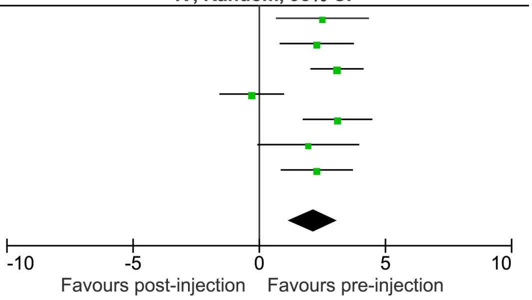

VII

Study or Subgroup	post-injection Mean	SD	Total	pre-injection Mean	SD	Total	Weight	Mean Difference IV, Random, 95% CI
Demirel et al.	14.21	2.9	202	15.11	2.8	202	8.0%	-0.90 [-1.46, -0.34]
Gismondi et al.	16.1	3	54	16.3	3	54	5.5%	-0.20 [-1.33, 0.93]
Goktas et al.	13.3	2.4	31	14.5	2.3	31	5.3%	-1.20 [-2.37, -0.03]
Karakurt et al. - aflibercept	14.83	2.68	53	15.64	3.18	53	5.5%	-0.81 [-1.93, 0.31]
Karakurt et al. - ranibizumab	15.83	3.2	81	15.14	2.95	81	6.3%	0.69 [-0.26, 1.64]
Kernt et al.	12.7	3.1	45	13.9	2.6	45	5.3%	-1.20 [-2.38, -0.02]
Kim, GN et al. - bevacizumab	14.5	2.28	19	13.67	2.16	19	4.4%	0.83 [-0.58, 2.24]
Kim, GN et al. - ranibizumab	14.21	2.83	10	14.08	2.48	10	2.3%	0.13 [-2.20, 2.46]
Lee, JW et al.	15	4.21	65	16.66	3.5	65	4.7%	-1.66 [-2.99, -0.33]
Lee, K et al. - hypertensive group	11.99	2.6	61	13.11	2.31	61	6.6%	-1.12 [-1.99, -0.25]
Lee, K et al. - nonhypertensive group	10.82	2.63	74	12.77	2.97	74	6.5%	-1.95 [-2.85, -1.05]
Omay et al. - bevacizumab	14.32	4.58	97	16.77	4.47	97	4.9%	-2.45 [-3.72, -1.18]
Omay et al. - ranibizumab	12.25	2.13	12	14.08	2.77	12	3.0%	-1.83 [-3.81, 0.15]
Ozkan et al.	14.8	2.7	21	15.6	3.2	21	3.4%	-0.80 [-2.59, 0.99]
Sengul et al. - 10/20 injections	16.35	2.38	62	16.04	2.07	62	7.0%	0.31 [-0.48, 1.10]
Sengul et al. - 20+ injections	15.95	2.48	44	15.79	2.32	44	6.0%	0.16 [-0.84, 1.16]
Sengul et al. - 3/10 injections	16.26	2.11	62	16.05	2.13	62	7.2%	0.21 [-0.54, 0.96]
Song, S. et al.	15.48	2.92	203	15.82	2.57	203	8.1%	-0.34 [-0.88, 0.20]
**Total (95% CI)**			**1196**			**1196**	**100.0%**	**-0.63 [-1.04, -0.22]**

Heterogeneity: Tau² = 0.47; Chi² = 49.87, df = 17 (P < 0.0001); I² = 66%
Test for overall effect: Z = 3.03 (P = 0.002)

Mean Difference IV, Random, 95% CI

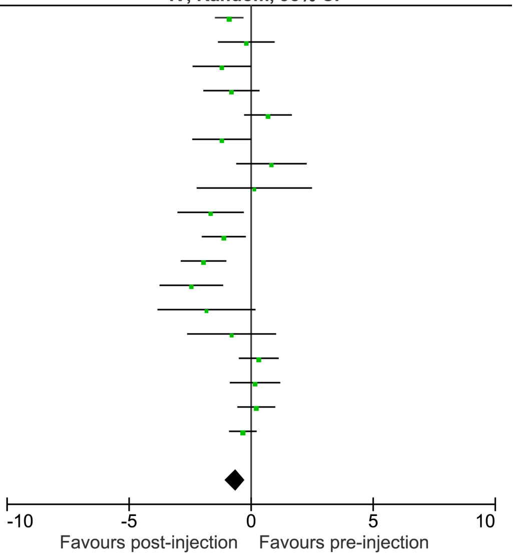

VIII

Study or Subgroup	post-injection Mean	SD	Total	pre-injection Mean	SD	Total	Weight	Mean Difference IV, Random, 95% CI
Kim, GN et al. - bevacizumab	15.36	1.85	19	13.67	2.16	19	10.9%	1.69 [0.41, 2.97]
Kim, GN et al. - ranibizumab	13.5	2.54	10	14.08	2.48	10	7.1%	-0.58 [-2.78, 1.62]
Kim, JY et al.	12.5	2.63	22	13.09	2.07	22	10.3%	-0.59 [-1.99, 0.81]
Lee, JW et al.	15.9	3.63	65	16.66	3.5	65	11.1%	-0.76 [-1.99, 0.47]
Lee, K et al. - hypertensive group	12.16	2.31	61	13.11	2.31	61	12.9%	-0.95 [-1.77, -0.13]
Lee, K et al. - nonhypertensive group	11.71	2.47	74	12.77	2.97	74	12.7%	-1.06 [-1.94, -0.18]
Omay et al. - bevacizumab	14.49	4.31	97	16.77	4.47	97	11.1%	-2.28 [-3.52, -1.04]
Omay et al. - ranibizumab	11.92	2.74	12	14.08	2.77	12	7.1%	-2.16 [-4.36, 0.04]
Ozkan et al.	16.4	3.3	21	15.6	3.2	21	8.0%	0.80 [-1.17, 2.77]
Totan et al.	18.4	3.7	30	16.5	3.3	30	8.7%	1.90 [0.13, 3.67]
**Total (95% CI)**			**411**			**411**	**100.0%**	**-0.44 [-1.25, 0.38]**

Heterogeneity: Tau² = 1.17; Chi² = 32.73, df = 9 (P = 0.0001); I² = 73%
Test for overall effect: Z = 1.05 (P = 0.29)

Mean Difference IV, Random, 95% CI

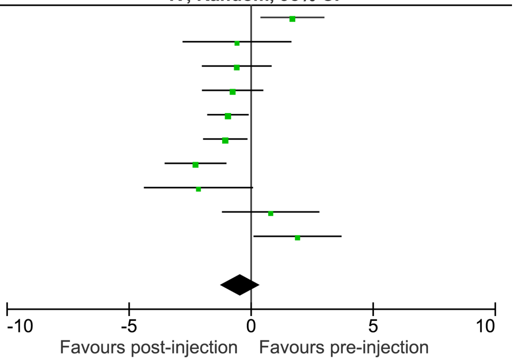

IX

Study or Subgroup	post-injection Mean	SD	Total	pre-injection Mean	SD	Total	Weight	Mean Difference IV, Random, 95% CI
Ding et al.	13.2	2.87	16	12.2	2	16	17.5%	1.00 [-0.71, 2.71]
Guler et al.	14.29	3.91	43	14.32	4.25	43	17.4%	-0.03 [-1.76, 1.70]
Kernt et al.	12.7	3	45	13.9	2.6	45	21.9%	-1.20 [-2.36, -0.04]
Lemos et al.	16.86	3.62	106	15.31	3.902	106	23.1%	1.55 [0.54, 2.56]
Song, J.H. et al.	15.4	2.75	38	16.1	3.34	38	20.2%	-0.70 [-2.08, 0.68]
**Total (95% CI)**			**248**			**248**	**100.0%**	**0.12 [-1.05, 1.29]**

Heterogeneity: Tau² = 1.28; Chi² = 14.96, df = 4 (P = 0.005); I² = 73%
Test for overall effect: Z = 0.21 (P = 0.84)

Mean Difference
IV, Random, 95% CI

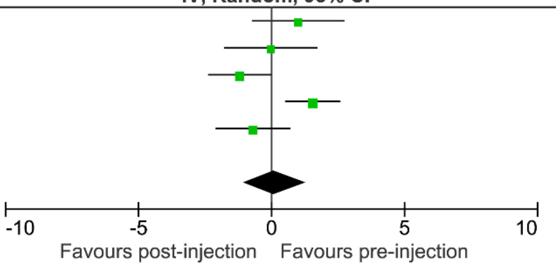

X

Study or Subgroup	post-injection Mean	SD	Total	pre-injection Mean	SD	Total	Weight	Mean Difference IV, Fixed, 95% CI
Ding et al.	13.22	2.94	16	12.2	2	16	2.5%	1.02 [-0.72, 2.76]
Gado et al.	17	1.598	30	17	1.839	30	10.2%	0.00 [-0.87, 0.87]
Guler et al.	13.23	4.37	43	14.32	4.25	43	2.3%	-1.09 [-2.91, 0.73]
Kaldirim et al - aflibercept	17.15	0.87	20	16.9	1.07	20	21.1%	0.25 [-0.35, 0.85]
Kaldirim et al. - ranibizumab	16.13	0.99	22	16.09	1.27	22	17.0%	0.04 [-0.63, 0.71]
Karakurt et al. - aflibercept	15.04	2.01	53	15.64	3.18	53	7.5%	-0.60 [-1.61, 0.41]
Karakurt et al. - ranibizumab	15.23	2.16	81	15.14	2.95	81	12.2%	0.09 [-0.71, 0.89]
Kim, GN et al. - bevacizumab	14.86	2.73	19	13.67	2.16	19	3.2%	1.19 [-0.38, 2.76]
Kim, GN et al. - ranibizumab	14.08	2.48	10	14.08	2.48	10	1.6%	0.00 [-2.17, 2.17]
Kim, JY et al.	12.23	2.72	22	13.09	2.07	22	3.8%	-0.86 [-2.29, 0.57]
Omay et al. - bevacizumab	15.19	4.68	97	16.77	4.47	97	4.7%	-1.58 [-2.87, -0.29]
Omay et al. - ranibizumab	14	4.15	12	14.08	2.77	12	1.0%	-0.08 [-2.90, 2.74]
Ozkan et al.	16.3	2.6	21	15.6	3.2	21	2.5%	0.70 [-1.06, 2.46]
Rensch et al.	14.5	3.2	25	14.2	3.2	25	2.5%	0.30 [-1.47, 2.07]
Song, J.H. et al.	16	2.86	38	16.1	3.34	38	3.9%	-0.10 [-1.50, 1.30]
Totan et al.	19.5	5.3	30	16.5	3.3	30	1.5%	3.00 [0.77, 5.23]
Tsai et al.	15.3	3	23	14.4	3.1	23	2.5%	0.90 [-0.86, 2.66]
**Total (95% CI)**			**562**			**562**	**100.0%**	**0.05 [-0.23, 0.32]**

Heterogeneity: Chi² = 22.71, df = 16 (P = 0.12); I² = 30%
Test for overall effect: Z = 0.33 (P = 0.74)

Mean Difference
IV, Fixed, 95% CI

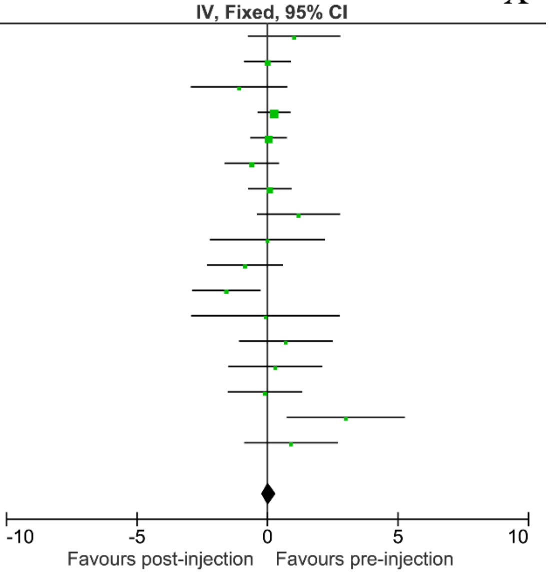

XI

Study or Subgroup	post-injection Mean	SD	Total	pre-injection Mean	SD	Total	Weight	Mean Difference IV, Fixed, 95% CI
Gado et al.	17	1.598	30	17	1.839	30	26.3%	0.00 [-0.87, 0.87]
Kim, JY et al.	12.55	2.09	22	13.09	2.07	22	13.2%	-0.54 [-1.77, 0.69]
Lee, K et al. - hypertensive group	12.75	2.51	61	13.11	2.31	61	27.2%	-0.36 [-1.22, 0.50]
Lee, K et al. - nonhypertensive group	12.55	3.09	74	12.77	2.97	74	20.9%	-0.22 [-1.20, 0.76]
Song, J.H. et al.	16.4	2.2	38	16.1	3.34	38	12.3%	0.30 [-0.97, 1.57]
**Total (95% CI)**			**225**			**225**	**100.0%**	**-0.18 [-0.63, 0.27]**

Heterogeneity: Chi² = 1.22, df = 4 (P = 0.88); I² = 0%
Test for overall effect: Z = 0.78 (P = 0.43)

Mean Difference
IV, Fixed, 95% CI

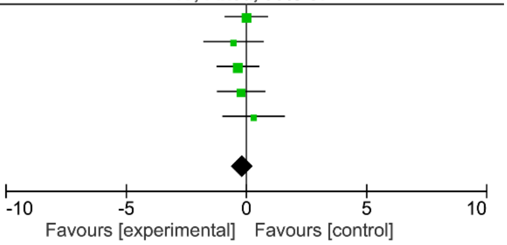

XII

Study or Subgroup	post-injection Mean	SD	Total	pre-injection Mean	SD	Total	Weight	Mean Difference IV, Fixed, 95% CI
Ding et al.	13.62	2.91	16	12.2	2	16	2.2%	1.42 [-0.31, 3.15]
Gado et al.	16	1.343	30	17	1.839	30	9.8%	-1.00 [-1.81, -0.19]
Kaldirim et al - aflibercept	17.05	0.88	20	16.9	1.07	20	17.6%	0.15 [-0.46, 0.76]
Kaldirim et al. - ranibizumab	16.32	0.99	22	16.09	1.27	22	14.3%	0.23 [-0.44, 0.90]
Kernt et al.	14.7	2.9	45	13.9	2.6	45	5.0%	0.80 [-0.34, 1.94]
Kim, JY et al.	12	2.78	22	13.09	2.07	22	3.1%	-1.09 [-2.54, 0.36]
Lee, K et al. - hypertensive group	13.1	2.4	61	13.11	2.31	61	9.3%	-0.01 [-0.85, 0.83]
Lee, K et al. - nonhypertensive group	12.79	3.4	74	12.77	2.97	74	6.1%	0.02 [-1.01, 1.05]
Maray et al.	15.13	2.21	30	15.47	2.93	30	3.8%	-0.34 [-1.65, 0.97]
Ozkan et al.	15.3	2.8	21	15.6	3.2	21	2.0%	-0.30 [-2.12, 1.52]
Ramezani et al.	12.8	2	43	13.3	2.7	43	6.4%	-0.50 [-1.50, 0.50]
Rensch et al.	14.2	2.8	25	14.2	3.2	25	2.3%	0.00 [-1.67, 1.67]
Soheilian et al.	14.7	1.6	44	14.2	2	44	11.3%	0.50 [-0.26, 1.26]
Tas et al.	13.72	3.04	40	13.31	3.22	40	3.4%	0.41 [-0.96, 1.78]
Totan et al.	18.5	4.8	30	16.5	3.3	30	1.5%	2.00 [-0.08, 4.08]
Tsai et al.	14.7	3.6	23	14.4	3.1	23	1.7%	0.30 [-1.64, 2.24]
**Total (95% CI)**			**546**			**546**	**100.0%**	**0.05 [-0.20, 0.31]**

Heterogeneity: Chi² = 19.92, df = 15 (P = 0.17); I² = 25%
Test for overall effect: Z = 0.42 (P = 0.68)

Mean Difference
IV, Fixed, 95% CI

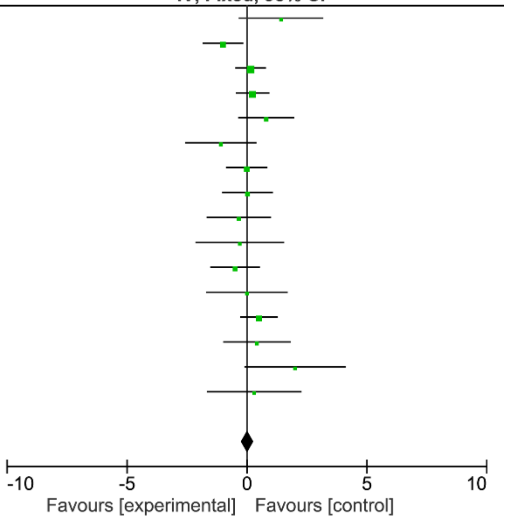

XIII

Study or Subgroup	post-injection Mean	SD	Total	pre-injection Mean	SD	Total	Weight	Mean Difference IV, Fixed, 95% CI
Gado et al.	16	1.623	30	17	1.839	30	27.3%	-1.00 [-1.88, -0.12]
Lee, K et al. - hypertensive group	13.22	2.71	61	13.11	2.31	61	26.4%	0.11 [-0.78, 1.00]
Lee, K et al. - nonhypertensive group	12.78	2.58	74	12.77	2.97	74	26.2%	0.01 [-0.89, 0.91]
Ramezani et al.	13.3	2.1	43	13.3	2.7	43	20.1%	0.00 [-1.02, 1.02]
**Total (95% CI)**			**208**			**208**	**100.0%**	**-0.24 [-0.70, 0.22]**

Heterogeneity: Chi² = 3.98, df = 3 (P = 0.26); I² = 25%
Test for overall effect: Z = 1.03 (P = 0.30)

Mean Difference
IV, Fixed, 95% CI

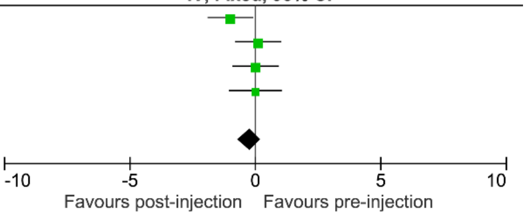

IXV

Study or Subgroup	post-injection Mean	SD	Total	pre-injection Mean	SD	Total	Weight	Mean Difference IV, Fixed, 95% CI
Ding et al.	13.84	3.08	16	12.2	2	16	2.0%	1.64 [-0.16, 3.44]
Gado et al.	16	2.024	30	17	1.839	30	6.8%	-1.00 [-1.98, -0.02]
Jaffar et al.	14.21	3.12	100	13.8	3.13	100	8.7%	0.41 [-0.46, 1.28]
Jo et al.	16.2	2.8	24	15.3	2.6	24	2.8%	0.90 [-0.63, 2.43]
Kaldirim et al - aflibercept	16.75	0.78	20	16.9	1.07	20	19.4%	-0.15 [-0.73, 0.43]
Kaldirim et al. - ranibizumab	16.4	0.91	22	16.09	1.27	22	15.3%	0.31 [-0.34, 0.96]
Kim, D et al.	13.61	3.05	83	14.11	2.76	83	8.3%	-0.50 [-1.38, 0.38]
Kim, JY et al.	12.32	2.92	22	13.09	2.07	22	2.9%	-0.77 [-2.27, 0.73]
Lee, K et al. - hypertensive group	13.2	2.01	61	13.11	2.31	61	11.1%	0.09 [-0.68, 0.86]
Lee, K et al. - nonhypertensive group	13.06	2.26	74	12.77	2.97	74	9.0%	0.29 [-0.56, 1.14]
Ozkan et al.	15.8	2.8	21	15.6	3.2	21	2.0%	0.20 [-1.62, 2.02]
Ramezani et al.	12.3	2.3	43	13.3	2.7	43	5.8%	-1.00 [-2.06, 0.06]
Rensch et al.	14	3.1	25	14.2	3.2	25	2.1%	-0.20 [-1.95, 1.55]
Totan et al.	17.1	4.1	30	16.5	3.3	30	1.8%	0.60 [-1.28, 2.48]
Tsai et al.	14	3.4	23	14.4	3.1	23	1.8%	-0.40 [-2.28, 1.48]
**Total (95% CI)**			**594**			**594**	**100.0%**	**-0.04 [-0.29, 0.22]**

Heterogeneity: Chi² = 17.25, df = 14 (P = 0.24); I² = 19%
Test for overall effect: Z = 0.30 (P = 0.77)

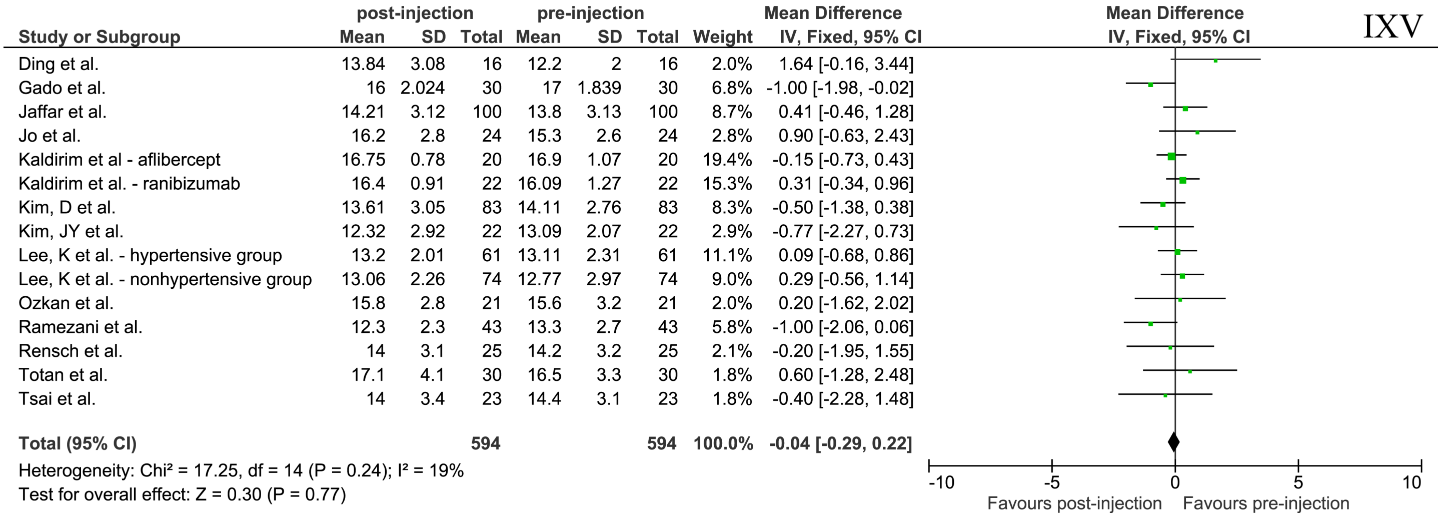

XV

Study or Subgroup	post-injection Mean	SD	Total	pre-injection Mean	SD	Total	Weight	Mean Difference IV, Fixed, 95% CI
Higashiyama et al.	13.9	2.7	22	13.7	3	22	10.5%	0.20 [-1.49, 1.89]
Jaffar et al.	13.79	3.07	100	13.8	3.16	100	40.1%	-0.01 [-0.87, 0.85]
Jo et al.	16.4	2.7	24	15.3	2.6	24	13.3%	1.10 [-0.40, 2.60]
Kim, D et al.	13.74	3.21	83	14.11	2.76	83	36.1%	-0.37 [-1.28, 0.54]
**Total (95% CI)**			**229**			**229**	**100.0%**	**0.03 [-0.52, 0.58]**

Heterogeneity: Chi² = 2.74, df = 3 (P = 0.43); I² = 0%
Test for overall effect: Z = 0.11 (P = 0.91)

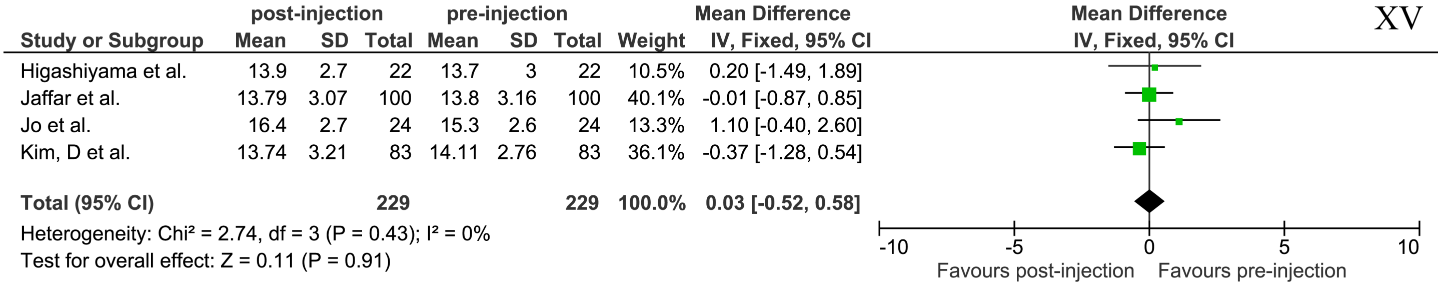

Black diamonds indicate the overall weighted mean difference (WMD). The size of the green box is inversely proportional to the variance. Horizontal lines indicate 95% CI. The vertical line in each panel shows the value for no effect (WMD = 0).

Anti-VEGF = anti vascular endothelial growth factor; IOP = intra-ocular pressure; CI = confidence interval; SD = standard deviation; I = 1 minute after injection; II = 5 minutes; III = 10 minutes; IV = 15 minutes; V = 30 minutes; VI = 1 hour; VII = 1 day; VIII = 1 week; IX = 2 weeks; X = 1 month; XI = 2 months; XII = 3 months; XIII = 4 months; IXV = 6 months; XV = 12 months.

FIGURE S6. Meta-analyses for the association of dexamethasone implant and IOP.

I

Study or Subgroup	post-injection Mean	post-injection SD	post-injection Total	pre-injection Mean	pre-injection SD	pre-injection Total	Weight	Mean Difference IV, Random, 95% CI
Alagöz et al.	16	2	34	16.5	3.4	34	17.9%	-0.50 [-1.83, 0.83]
Bullut et al.	18.7	2.2	33	17.6	1.7	33	20.6%	1.10 [0.15, 2.05]
Karakurt et al. - dexamethasone implant	16.09	2.38	54	16.31	3.13	54	19.9%	-0.22 [-1.27, 0.83]
Mazzarella et al. - non-glaucoma group	12.1	5.4	65	14.1	3	65	16.7%	-2.00 [-3.50, -0.50]
Mazzarella et al. - OHT/glaucoma group (treated)	19.9	11.2	27	15.1	3.8	27	4.8%	4.80 [0.34, 9.26]
Özata et al.	16.64	2.738	50	15.54	2.476	50	20.1%	1.10 [0.08, 2.12]
**Total (95% CI)**			**263**			**263**	**100.0%**	**0.21 [-0.88, 1.30]**

Heterogeneity: Tau² = 1.26; Chi² = 20.34, df = 5 (P = 0.001); I² = 75%
Test for overall effect: Z = 0.38 (P = 0.70)

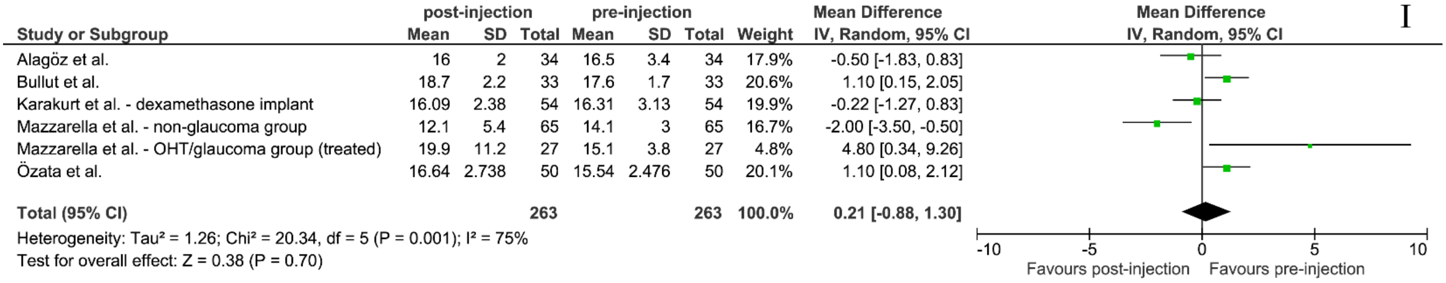

II

Study or Subgroup	post-injection Mean	post-injection SD	post-injection Total	pre-injection Mean	pre-injection SD	pre-injection Total	Weight	Mean Difference IV, Fixed, 95% CI
Ali et al.	15.25	2.63	30	14.85	2.4	30	7.0%	0.40 [-0.87, 1.67]
Bullut et al.	18.5	2.2	33	17.6	1.7	33	12.6%	0.90 [-0.05, 1.85]
Choi et al.	14.31	4.1	540	13.45	2.95	540	62.4%	0.86 [0.43, 1.29]
Cunha et al.	15.7	1	10	15	0.8	10	18.0%	0.70 [-0.09, 1.49]
**Total (95% CI)**			**613**			**613**	**100.0%**	**0.80 [0.47, 1.14]**

Heterogeneity: Chi² = 0.56, df = 3 (P = 0.91); I² = 0%
Test for overall effect: Z = 4.68 (P < 0.00001)

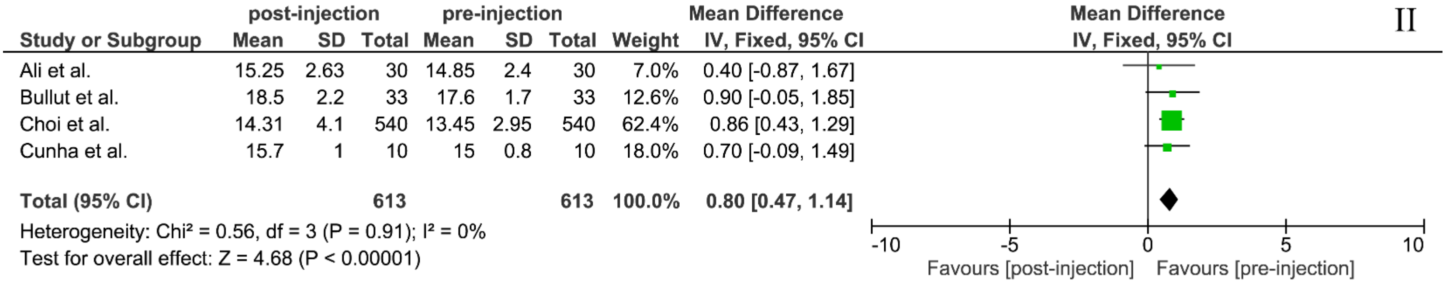

III

Study or Subgroup	post-injection Mean	post-injection SD	post-injection Total	pre-injection Mean	pre-injection SD	pre-injection Total	Weight	Mean Difference IV, Random, 95% CI
Albo-Linero et al.	15.82	6.73	79	13.7	4.36	79	4.6%	2.12 [0.35, 3.89]
Arikan Yogun et al.	17.9	4.3	41	15	2	41	5.0%	2.90 [1.45, 4.35]
Arrascue et al.	17.97	5.4	37	14.15	2.8	37	4.3%	3.82 [1.86, 5.78]
Bellocq et al. (2015)	15.3	4.6	50	12.3	2.6	50	5.0%	3.00 [1.54, 4.46]
Bellocq et al. (2017)	15.5	6	100	13.4	3.9	100	5.1%	2.10 [0.70, 3.50]
Bullut et al.	18.4	2.5	33	17.6	1.7	33	5.5%	0.80 [-0.23, 1.83]
Choi et al.	15.94	4.63	540	13.45	2.95	540	6.1%	2.49 [2.03, 2.95]
Coskun et al.	20.2	8.4	17	14.9	3.4	17	2.0%	5.30 [0.99, 9.61]
Cunha et al.	14.1	0.6	10	15	0.8	10	5.9%	-0.90 [-1.52, -0.28]
Frère et al.	18	6	20	16	5	20	2.6%	2.00 [-1.42, 5.42]
Gado et al.	18	3.505	30	16	2.769	30	4.8%	2.00 [0.40, 3.60]
Kaldirim et al. - dexamethasone implant	16.8	0.69	20	16.5	0.83	20	6.1%	0.30 [-0.17, 0.77]
Karakurt et al. - dexamethasone implant	15.31	2.05	54	16.31	3.13	54	5.6%	-1.00 [-2.00, -0.00]
Mazzarella et al. - non-glaucoma group	17.5	5.4	65	14.1	3	65	4.9%	3.40 [1.90, 4.90]
Mazzarella et al. - OHT/glaucoma group (treated)	19.7	8.1	27	15.1	3.8	27	2.7%	4.60 [1.23, 7.97]
Nalcaci et al.	16.4	3.9	20	14.9	2.7	20	4.1%	1.50 [-0.58, 3.58]
Pohlmann et al.	16.3	4.9	109	14	3.7	109	5.4%	2.30 [1.15, 3.45]
Unsal et al.	18.91	4.63	46	16.47	3.1	46	4.8%	2.44 [0.83, 4.05]
Yorgun et al.	17.9	4.3	41	15	2	41	5.0%	2.90 [1.45, 4.35]
Zarranz-Ventura et al.	18	7	146	14.1	4	146	5.2%	3.90 [2.59, 5.21]
Özata et al.	17.08	2.988	50	15.54	2.476	50	5.5%	1.54 [0.46, 2.62]
**Total (95% CI)**			**1535**			**1535**	**100.0%**	**2.04 [1.31, 2.78]**

Heterogeneity: Tau² = 2.26; Chi² = 170.63, df = 20 (P < 0.00001); I² = 88%
Test for overall effect: Z = 5.46 (P < 0.00001)

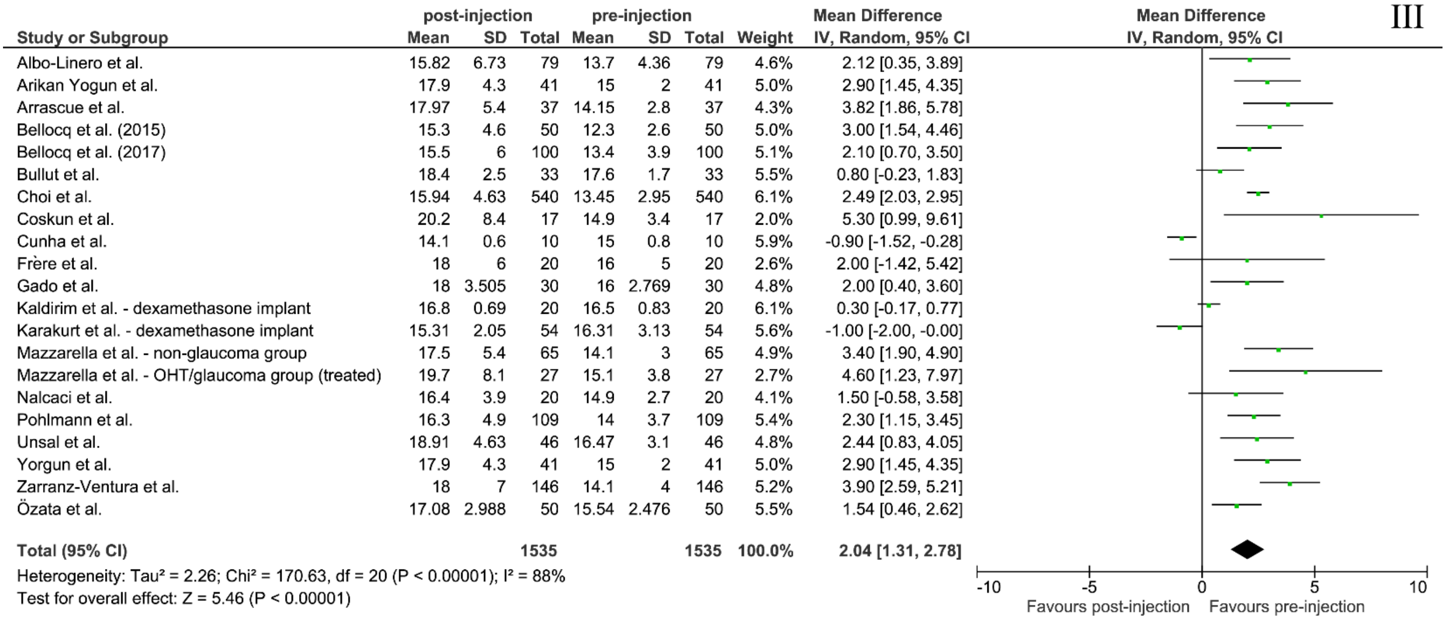

IV

Study or Subgroup	post-injection Mean	post-injection SD	post-injection Total	pre-injection Mean	pre-injection SD	pre-injection Total	Weight	Mean Difference IV, Random, 95% CI
Arrascue et al.	15.82	4	37	14.15	2.8	37	12.3%	1.67 [0.10, 3.24]
Bellocq et al. (2017)	14.2	3.1	100	13.4	3.9	100	15.3%	0.80 [-0.18, 1.78]
Choi et al.	16.85	5.96	540	13.45	2.95	540	17.0%	3.40 [2.84, 3.96]
Gado et al.	18	2.059	30	16	2.769	30	14.0%	2.00 [0.77, 3.23]
Mazzarella et al. - non-glaucoma group	18.4	6.6	65	14.1	3	65	11.4%	4.30 [2.54, 6.06]
Mazzarella et al. - OHT/glaucoma group (treated)	20.9	7.8	27	15.1	3.8	27	6.0%	5.80 [2.53, 9.07]
Nalcaci et al.	17.1	4.4	20	14.9	2.7	20	9.2%	2.20 [-0.06, 4.46]
Özata et al.	17.3	2.97	50	15.54	2.476	50	14.8%	1.76 [0.69, 2.83]
**Total (95% CI)**			**869**			**869**	**100.0%**	**2.49 [1.51, 3.46]**

Heterogeneity: Tau² = 1.38; Chi² = 32.84, df = 7 (P < 0.0001); I² = 79%
Test for overall effect: Z = 4.99 (P < 0.00001)

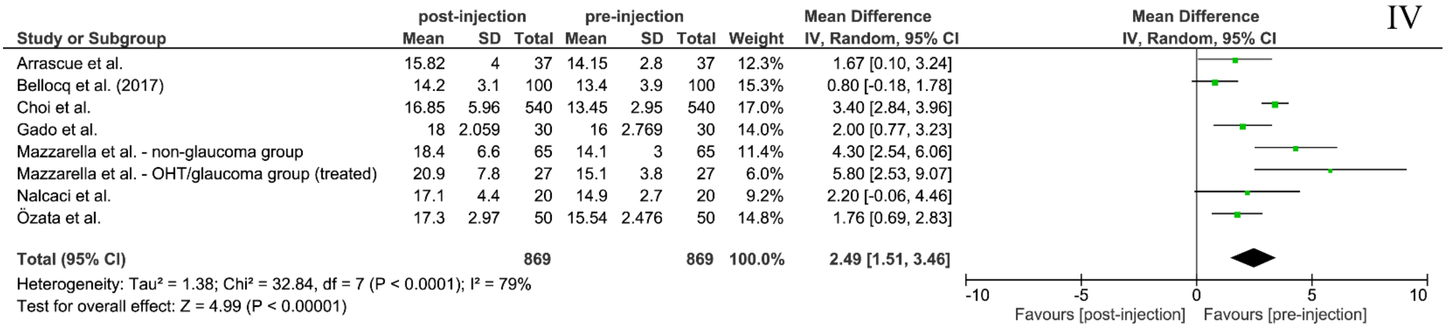

V

Study or Subgroup	post-injection Mean	SD	Total	pre-injection Mean	SD	Total	Weight	Mean Difference IV, Random, 95% CI
Albo-Linero et al.	15.56	6.26	79	13.7	4.36	79	4.3%	1.86 [0.18, 3.54]
Arikan Yogun et al.	18.4	5.1	41	15	2	41	4.3%	3.40 [1.72, 5.08]
Bansal et al.	15.9	5.2	67	13.4	2.9	67	5.3%	2.50 [1.07, 3.93]
Bullut et al.	18.1	1.6	33	17.6	1.7	33	9.1%	0.50 [-0.30, 1.30]
Choi et al.	15.15	5.07	540	13.45	2.95	540	11.3%	1.70 [1.21, 2.19]
Coskun et al.	14.9	5.7	17	14.9	3.4	17	1.6%	0.00 [-3.15, 3.15]
Cunha et al.	15.6	0.9	10	15	0.8	10	9.4%	0.60 [-0.15, 1.35]
Gado et al.	19	1.792	30	16	2.769	30	6.6%	3.00 [1.82, 4.18]
Kaldirim et al. - dexamethasone implant	18.2	0.83	20	16.5	0.83	20	11.1%	1.70 [1.19, 2.21]
Mazzarella et al. - non-glaucoma group	15	3.5	65	14.1	3	65	6.9%	0.90 [-0.22, 2.02]
Mazzarella et al. - OHT/glaucoma group (treated)	17.4	6.9	27	15.1	3.8	27	1.8%	2.30 [-0.67, 5.27]
Nalcaci et al.	15.6	3.6	20	14.9	2.7	20	3.5%	0.70 [-1.27, 2.67]
Pohlmann et al.	15	5.6	109	14	3.7	109	6.1%	1.00 [-0.26, 2.26]
Unsal et al.	18.43	2.44	46	16.47	3.1	46	6.8%	1.96 [0.82, 3.10]
Yorgun et al.	18.4	5.1	41	15	2	41	4.3%	3.40 [1.72, 5.08]
Zarranz-Ventura et al.	15.9	5	146	14.1	4	146	7.4%	1.80 [0.76, 2.84]
**Total (95% CI)**			**1291**			**1291**	**100.0%**	**1.65 [1.22, 2.07]**

Heterogeneity: Tau² = 0.36; Chi² = 35.19, df = 15 (P = 0.002); I² = 57%
Test for overall effect: Z = 7.57 (P < 0.00001)

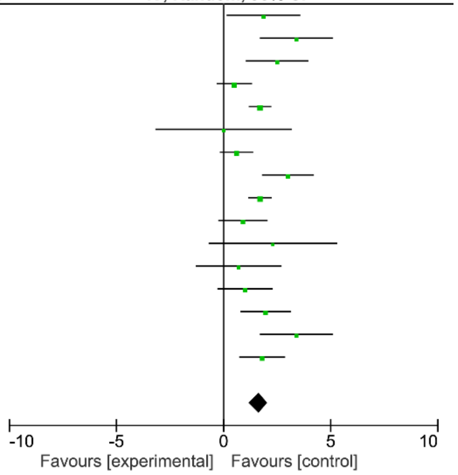

VI

Study or Subgroup	post-injection Mean	SD	Total	pre-injection Mean	SD	Total	Weight	Mean Difference IV, Fixed, 95% CI
Frère et al.	15	4	20	16	5	20	4.4%	-1.00 [-3.81, 1.81]
Gado et al.	19	1.134	30	16	2.769	30	30.1%	3.00 [1.93, 4.07]
Nalcaci et al.	16.2	4.2	20	14.9	2.7	20	7.2%	1.30 [-0.89, 3.49]
Unsal et al.	16.65	2.3	46	16.47	3.1	46	27.7%	0.18 [-0.94, 1.30]
Özata et al.	15.9	2.922	50	15.54	2.476	50	30.6%	0.36 [-0.70, 1.42]
**Total (95% CI)**			**166**			**166**	**100.0%**	**1.11 [0.53, 1.70]**

Heterogeneity: Chi² = 18.76, df = 4 (P = 0.0009); I² = 79%
Test for overall effect: Z = 3.71 (P = 0.0002)

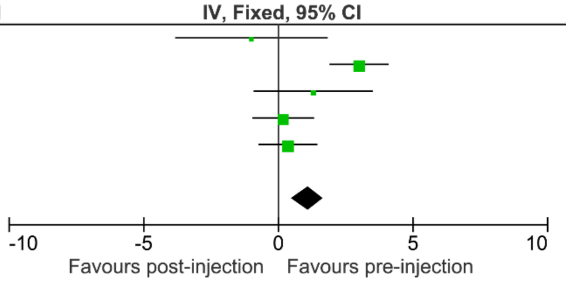

VII

Study or Subgroup	post-injection Mean	SD	Total	pre-injection Mean	SD	Total	Weight	Mean Difference IV, Random, 95% CI
Albo-Linero et al.	14.7	5.54	79	13.7	4.36	79	4.0%	1.00 [-0.55, 2.55]
Arikan Yogun et al.	15.8	3.4	41	15	2	41	5.2%	0.80 [-0.41, 2.01]
Arrascue et al.	15.38	3	37	14.15	2.8	37	4.8%	1.23 [-0.09, 2.55]
Ayar et al.	16.5	4.2	26	15.7	2.9	26	3.0%	0.80 [-1.16, 2.76]
Bansal et al.	17	5.3	67	13.4	2.9	67	4.4%	3.60 [2.15, 5.05]
Bellocq et al. (2017)	13.4	4.2	100	13.4	3.9	100	5.5%	0.00 [-1.12, 1.12]
Bullut et al.	18.1	1.8	33	17.6	1.7	33	6.6%	0.50 [-0.34, 1.34]
Choi et al.	14.1	4.08	540	13.45	2.95	540	8.3%	0.65 [0.23, 1.07]
Coskun et al.	14.5	3.2	17	14.9	3.4	17	2.6%	-0.40 [-2.62, 1.82]
Cunha et al.	18.2	1.8	10	15	0.8	10	5.1%	3.20 [1.98, 4.42]
Kaldirim et al. - dexamethasone implant	18.3	0.86	20	16.5	0.83	20	7.9%	1.80 [1.28, 2.32]
Mazzarella et al. - non-glaucoma group	13.9	3.6	65	14.1	3	65	5.4%	-0.20 [-1.34, 0.94]
Mazzarella et al. - OHT/glaucoma group (treated)	16.7	6.1	27	15.1	3.8	27	1.9%	1.60 [-1.11, 4.31]
Nalcaci et al.	15.5	2.3	20	14.9	2.7	20	4.0%	0.60 [-0.95, 2.15]
Pohlmann et al.	14.6	3.2	109	14	3.7	109	6.3%	0.60 [-0.32, 1.52]
Ryder et al.	16.62	5.97	22	14.68	3.02	22	1.8%	1.94 [-0.86, 4.74]
Unsal et al.	16.56	2.1	46	16.47	3.1	46	5.6%	0.09 [-0.99, 1.17]
Yorgun et al.	15.8	3.4	41	15	2	41	5.2%	0.80 [-0.41, 2.01]
Zarranz-Ventura et al.	14.4	4	146	14.1	4	146	6.3%	0.30 [-0.62, 1.22]
Özata et al.	15.88	2.576	50	15.54	2.476	50	6.0%	0.34 [-0.65, 1.33]
**Total (95% CI)**			**1496**			**1496**	**100.0%**	**0.91 [0.49, 1.33]**

Heterogeneity: Tau² = 0.50; Chi² = 54.59, df = 19 (P < 0.0001); I² = 65%
Test for overall effect: Z = 4.25 (P < 0.0001)

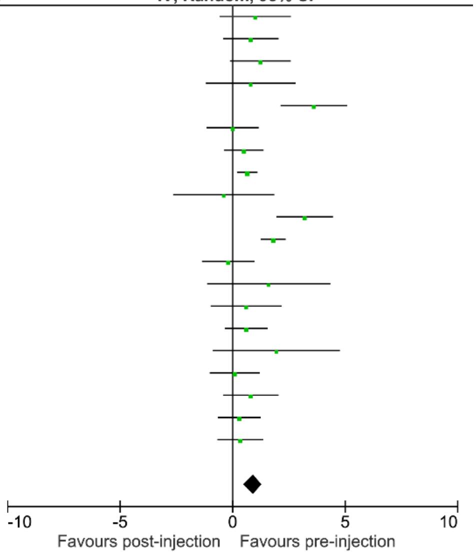

VIII

Study or Subgroup	post-injection Mean	SD	Total	pre-injection Mean	SD	Total	Weight	Mean Difference IV, Fixed, 95% CI
Albo-Linero et al.	14.25	5.4	79	13.7	4.36	79	4.6%	0.55 [-0.98, 2.08]
Bellocq et al. (2017)	14	4.7	100	13.4	3.9	100	7.5%	0.60 [-0.60, 1.80]
Choi et al.	13.8	4.04	540	13.45	2.95	540	60.2%	0.35 [-0.07, 0.77]
Coskun et al.	13.3	4.7	17	14.9	3.4	17	1.4%	-1.60 [-4.36, 1.16]
Mazzarella et al. - non-glaucoma group	13.1	3	65	14.1	3	65	10.1%	-1.00 [-2.03, 0.03]
Mazzarella et al. - OHT/glaucoma group (treated)	16.4	2.7	27	15.1	3.8	27	3.5%	1.30 [-0.46, 3.06]
Zarranz-Ventura et al.	14.6	4	146	14.1	4	146	12.7%	0.50 [-0.42, 1.42]
**Total (95% CI)**			**974**			**974**	**100.0%**	**0.27 [-0.06, 0.59]**

Heterogeneity: Chi² = 9.71, df = 6 (P = 0.14); I² = 38%
Test for overall effect: Z = 1.59 (P = 0.11)

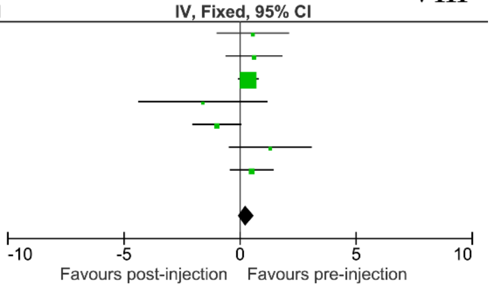

Black diamonds indicate the overall weighted mean difference (WMD). The size of the green box is inversely proportional to the variance. Horizontal lines indicate 95% CI. The vertical line in each panel shows the value for no effect (WMD = 0).
IOP = intra-ocular pressure; CI = confidence interval; SD = standard deviation; I = 1 day after injection; II = 1 week; III = 1 month; IV = 2 months; V = 3 months; VI = 4 months; VII = 6 months; VIII = 12 months.
